# Anti‐Inflammatory Effect of 21*α*‐Methylmelianol In Vitro and In Vivo via NF‐*κ* B/STAT Signaling Pathway Modulation

**DOI:** 10.1002/fsn3.71310

**Published:** 2025-12-04

**Authors:** Fan Cao, Hua Wu, Zhen He, Mengxin Di, Bin Liu, Zhenwei Chen, Jieming Xie, Yonghong Zhang, Xinhua Ma

**Affiliations:** ^1^ Fujian Provincial Key Laboratory of Natural Medicine Pharmacology, Department of Pharmacy Fujian Medical University Fuzhou China

**Keywords:** 21α‐methylmelianol, anti‐inflammatory, *Melia azedarach*, RAW264.7 cells, ulcerative colitis

## Abstract

*Melia azedarach* L., also known as the chinaberry tree, has long been used in traditional medicine to treat inflammatory conditions. In this study, we preliminarily investigated the anti‐inflammatory effect of 21*α*‐methylmelianol (MMN), a tirucallane‐type triterpenoid isolated from *M. azedarach* , in lipopolysaccharide (LPS)‐mediated RAW264.7 macrophages in vitro and in a dextran sodium sulfate (DSS)‐stimulated ulcerative murine colitis model in vivo. MMN reduced LPS‐stimulated nitric oxide production without inducing cytotoxicity. It significantly attenuated LPS‐induced protein expression of reactive oxygen species and effectively suppressed the production of pro‐inflammatory factors, including tumor necrosis factor‐alpha (TNF‐*α*), interleukin (IL)‐6, and IL‐1*β*. In addition, mechanistic investigations showed that MMN inhibited signal transduction and activator of STAT3 phosphorylation and activation of nuclear factor *κ*B (NF‐*κ*B). In mice with DSS‐induced colitis, MMN attenuated DSS‐mediated pathological manifestations, including colon shortening, body weight loss, and histopathological alterations. Its in vivo effects were associated with the regulation of pro‐ and anti‐inflammatory cytokine levels through suppression of the NF‐*κ*B signaling cascade and downstream inflammatory pathways. Collectively, these findings provide compelling evidence of the anti‐inflammatory properties of MMN and highlight its therapeutic potential for the management of inflammatory disorders.

## Graphical abstract

This study investigated the anti‐inflammatory effects of 21α‐methylmelianol (MMN), a tirucallane triterpenoid isolated from *Melia azedarach* , in lipopolysaccharide‐ stimulated RAW264.7 macrophages in vitro, and in a dextran sodium sulfate‐induced ulcerative murine colitis model in vivo.

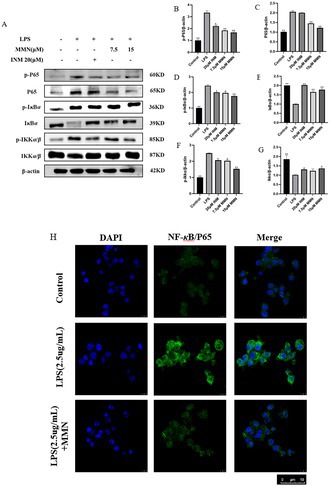

## 1 Introduction

*Melia azedarach* L., commonly known as chinaberry, is a plant of the Meliaceae family that is indigenous to the tropical and subtropical regions of Asia, including southern China (Mabberley 1984). In Vietnam and Japan, *M. azedarach* has traditionally been employed as a repellent, antifungal agent, insecticide, analgesic, antidiabetic, and dermatological remedy (Warrier et al. 1994). In various systems of traditional medicine, it has been used to treat diverse pathological conditions, particularly leprosy, scurvy, burn wounds, and malaria (Bohnenstengel et al. 1999). Phytochemical studies have identified a wide range of bioactive constituents from different parts of *M. azedarach* , including limonenes, triterpenoids, steroids, and flavonoids (Su et al. 2011; Akihisa et al. 2013; Zhou et al. 2016; Song et al. 2020). Among these, triterpenoids have shown multiple biological activities, such as cytotoxic (He et al. 2009; Ntalli et al. 2010; Yuan et al. 2013), antibacterial (Mehmood et al. 2013), antioxidant (Dias et al. 2022), antifeedant (Huang et al. 2021), insecticidal (Khoshraftar et al. 2019), and antiviral (Kim et al. 1999) effects. Traditionally, the fruit of *M. azedarach* has been used for its insecticidal, anti‐hemorrhoidal, antiseptic, antibacterial, and central nervous system inhibitory properties (Rastogi et al. 1990). In Korea, it is also applied in the treatment of dermatitis and rubella (Kim et al. 1999). To date, however, most studies have examined the bioactivity of *M. azedarach* crude extracts, and the specific contributions of individual bioactive components remain unclear.

Inflammation is the body's innate protective response to diverse external stimuli, including pathogen invasion, tissue injury, and irritants (Feng et al. 2021). However, uncontrolled inflammation can exert detrimental effects on the body and has been linked to the pathogenesis of several chronic disorders, including obesity, diabetes, rheumatoid arthritis, and bronchial asthma (Cao et al. 2021). Macrophages perform a key role in host defense mechanisms by actively combating pathogenic agents, and subsequently orchestrating the inflammatory response (Kim et al. 2019). RAW 264.7 macrophages have been extensively employed as models for investigating anti‐inflammatory mechanisms and evaluating potential therapeutic agents. During the inflammatory response, macrophages facilitate endocytosis of bacterial fragments, which subsequently triggers the generation of nitric oxide (NO) and proinflammatory cytokines. These mediators amplify the local inflammatory cascade (Yang et al. 2012). Activation of NF‐*κ*B and members of the mitogen‐activated protein kinase (MAPK) family (e.g., ERK and p38) performs a vital role in pro‐inflammatory cytokine production. These signaling pathways are triggered during lipopolysaccharide (LPS)‐induced macrophage inflammation (Cao et al. 2021). Consequently, they have been extensively studied as key molecular targets for anti‐inflammatory drug development.

Ulcerative colitis (UC) is a chronic inflammatory disease that primarily affects the colon. It predominantly manifests in 30–40‐year‐old adults and can result in significant disability (Høivik et al. 2013). The disease is characterized by recurrent and intermittent mucosal inflammation originating in the rectum and extending proximally to the colon. The primary therapeutic goal is to achieve and sustain clinical and endoscopic remission (Choi et al. 2023). Pharmacological management of UC primarily involves anti‐inflammatory medications, including corticosteroids and 5‐aminosalicylic acid (5‐ASA). However, these agents also have substantial side effects, including nausea, headache, and rash. Consequently, numerous studies have centered on investigating the potential of natural products, characterized by low toxicity and minimal adverse reactions, for the preventive and therapeutic treatment of UC (Wang et al. 2022).

Macrophages play a critical role in the immune system (Zhang et al. 2021). They differentiate from monocytes and function by phagocytosing and degrading cellular debris, pathogenic microorganisms, and various foreign substances (Hirayama et al. 2017). In colitis models, an increase in macrophage populations has been observed, accompanied by abnormal inflammatory activation in colon tissue (Ebihara et al. 2023). Activated macrophages release pro‐inflammatory factors and mediators that contribute to intestinal injury (Gao et al. 2021). Triterpenoids isolated from *M. azedarach* have been shown to inhibit macrophage‐mediated inflammatory responses induced by LPS, alleviate colitis symptoms, and enhance immunomodulatory function (Shen et al. 2022).

Phytochemical analyses have identified triterpenoids as the principal bioactive constituents of *M. azedarach* , including 21*α*‐methylmelianol (MMN), a monomeric tirucallane‐type triterpenoid. A previous study demonstrated that triterpenoids are ideal candidates for the exploitation of new anti‐inflammatory pharmaceuticals. In particular, pretreatment of LPS‐stimulated microglia with triterpenes markedly inhibits the formation of reactive oxygen species (ROS) and prevents elevations in NO, TNF‐*α*, and IL‐6 levels. Pretreatment with triterpenes significantly reduces inducible nitric oxide synthase (iNOS) and cyclooxygenase (COX)‐2 expression (Liu et al. 2020). Furthermore, disruption of the NF‐*κ*B signaling cascade significantly modulates the transcriptional regulation of genes involved in inflammatory responses and programmed cell death (Costa et al. 2012). To date, however, the prophylactic effect of MMN against DSS‐activated inflammatory colitis in murine models has not been fully elucidated. In this study, we investigated the anti‐inflammatory efficacy of MMN and explored its potential mechanisms using LPS‐stimulated macrophages and a DSS‐activated UC mouse model.

## 2 Material and Methods

### 2.1 Reagents

ROS detection kits were from BioTek (Shanghai, China). The ELISA kit for IL‐1*β*, TNF‐*α*, and IL‐6 assay was obtained from Meimian Biotechnology Ltd. (Shanghai, China). LPS was obtained from Sigma Aldrich Chemical Company (Missouri, USA), and 5‐ASA from Shanghai Aladdin Biochemical Co. DSS was obtained from MP Biomedicals (California, USA). Indomethacin was purchased from Aladdin Inc. (Connecticut, USA). Primary and secondary antibodies utilized in this study were obtained from Huabio (Hangzhou, China) and Abcam (Cambridge, UK), respectively. The NO, malondialdehyde (MDA), reduced glutathione (GSH), and superoxide dismutase (SOD) assay kits were purchased from Nanjing Jianjian Bioengineering Research Co (Nanjing). For in vitro studies, MMN was dissolved in DMSO (final concentration < 0.1%) with equivalent DMSO used for vehicle controls. For in vivo administration, MMN was suspended in 0.5% CMC‐Na/saline.

### 2.2 Isolation of MMN

*M. azedarach* fruit (26.5 kg), harvested in Fuzhou, Fujian Province, China, was finely ground into powder and macerated in methanol (MeOH) for 7 days. The resulting extract was partitioned with solvents of increasing polarity, including petroleum ether, dichloromethane (CH_2_Cl_2_), EtOAc, and *n*‐BuOH, to obtain distinct fractions for further analysis. The CH_2_Cl_2_ extract was eluted using a silica gel with a PE–EtOAc gradient, and 13 fractions (Fr.1–13) were obtained. Fr.5 was separated using an ODS gel column and compound **1** (925.8 mg) was isolated using Sephadex LH‐20 resins (MeOH) and HPLC (CH_3_CN/H_2_O).

**FIGURE 1:**
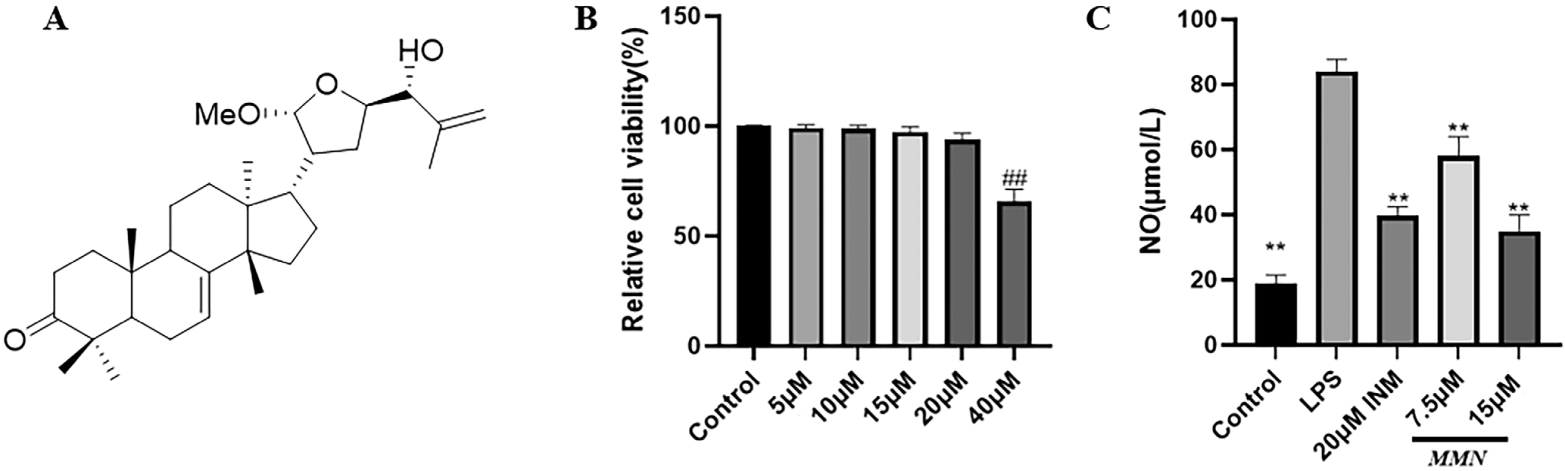
Effect of MMN on RAW 264.7 cell viability and LPS‐mediated NO production. (A) The structure of MMN (21α‐methylmelianol). (B) The cell viability was assayed by the SRB method. (C) NO production was examined by the Griess test. The data is the mean ± SD (*n* = 3). Significance was assessed by one‐way ANOVA using the Tukey test. **Significant at *p* < 0.01 compared to the LPS‐mediated group. ^##^Significant at *p* < 0.01 relative to the control group. INM (Indomethacin), as positive control.

Identification of 21*α*‐methylmelianol (MMN): Based on a HR‐ESI‐MS *m/z* of 507.3477 (calculated [M + Na]^+^, 507.3445), the molecular formula of compound **1** was found to be C_31_H_48_O_4_. ^1^H‐NMR (600 MHz, CDCl_3_): *δ*_H_5.31 (1H, d, *J* = 7.2, 6.8, H‐7), 5.00 (1H, s, H‐26), 4.90 (1H, s, H‐26), 4.80 (1H, d, H‐21), 4.06 (1H, m, H‐23), 3.93 (1H, td, H‐24), 3.35 (3H, s, H‐21OMe), 2.78 (1H, tt, *J* = 13.5, 3.8, 3.6, H‐2), 2.31 (1H, brs, H‐24OH), 2.31 (1H, dd, J = 13.3, 3.6, H‐9), 2.29 (1H, m, H‐9), 2.23 (1H, m, H‐20), 2.21 (1H, m, *J* = 14.2, 13.5, 3.6, H‐2), 2.08 (2H, m, H‐6), 1.99 (1H, m, *J* = 13.5, 3.8, 3.6, H‐1), 1.97 (2H, m, H‐22), 1.77 (1H, m, *J* = 13.4, 3.8, 3.6, H‐12), 1.73 (1H, m, H‐17), 1.72 (1H, m, *J* = 13.2, 3.6, H‐5), 1.71 (1H, m, *J* = 13.8, 13.4, 3.6, H‐12), 1.71 (3H, s, H‐27Me), 1.60 (2H, m, H‐11), 1.50 (2H, m, H‐15), 1.44 (1H, ddd, *J* = 14.2, 3.5, 4.6, H‐1), 1.11 (3H, s, H‐29Me), 1.04 (3H, s, H‐28Me), 1.01 (3H, s, H‐19Me), 1.01 (3H, s, H‐30Me), 0.85 (3H, s, H‐18Me). ^13^C‐NMR (150 MHz, CDCl_3_): *δ*_*C*_216.9 (C‐3), 145.7 (C‐8), 144.6 (C‐25), 118.3 (C‐7), 113.4 (C‐26), 108.9 (C‐21), 78.9 (C‐24), 78.4 (C‐23), 55.6 (C‐OMe), 52.5 (C‐5), 51.1 (C‐14), 50.6 (C‐14), 48.7 (C‐9), 48.5 (C‐20), 48.0 (C‐4), 43.8 (C‐13), 38.6 (C‐1), 35.2 (C‐10), 35.0 (C‐2), 34.8 (C‐23), 34.0 (C‐15), 31.8 (C‐12), 27.6 (C‐16), 27.4 (C‐30), 24.7 (C‐28), 24.5 (C‐6), 22.8 (C‐18), 21.7 (C‐29), 18.3 (C‐27), 17.9 (C‐11), 12.9 (C‐19), and its ^1^H and ^13^C NMR data were in agreement with 21*α*‐methylmelianol (MMN, Figure 1A; Figures S1–S3) (Yan et al. 2008). The purity of compound **1** was verified to be > 95% by HPLC (Figure S4).

### 2.3 Cell Culture

RAW 264.7 cells were obtained from Shanghai Cell Bank of the Chinese Academy of Science. They were incubated in Dulbecco's modified Eagle medium containing 10% fetal bovine serum (Gasparotto et al. 2013). Macrophages were incubated at 37°C in a humidified environment containing 5% CO_2_ for 2 h with MMN (DMSO, final concentration < 0.1%) and then activated using 2.5 μg/mL LPS within the designated time periods. The concentration of 2.5 μg/mL LPS was selected as it is well‐established in the literature to induce a robust inflammatory response in RAW264.7 cells, and our cytotoxicity assays confirmed that it does not significantly reduce cell viability (Chotphruethipong et al. 2023).

### 2.4 Determination of NO Production and Cell Viability Assays

Macrophages were seeded in 96‐well plates at a density of 5 × 10^5^/mL and cultured over 24 h. The experimental group was stimulated by fresh media supplemented with 2.5 μg/mL LPS and 5–40 μM MMN, followed by a 24‐h incubation. Nitrite accumulation in the culture supernatants was determined using Griess reagent as an indicator of NO generation. The sulforhodamine B assay was used to determine cell viability. To this end, the supernatant was removed, and then sulfanilamide B was added and stained at 25°C for 30 min. After washing away the staining solution, Tris solution was added to each well. For nitrite measurement, Griess reagent was mixed with the culture supernatant and incubated for 10 min in the dark. Absorbance was measured at 515 nm for cell viability and 540 nm for NO production (Vajrabhaya and Korsuwannawong 2018).

### 2.5 Measurement of Cytokine Production

The levels of TNF‐*α*, IL‐6, and IL‐1*β* pro‐inflammatory factors in mouse colon tissue and cultured cells were quantitatively analyzed by ELISA. Macrophages were inoculated in 96 well plates at a density of 2 × 10^4^/mL and cultured over 24 h (Huang et al. 1994). Macrophages were pretreated by MMN for 2 h and then cultured with 2.5 μg/mL LPS over 24 h. Subsequently, the cell culture supernatant was harvested and subjected to cytokine quantification by ELISA kits, following the manufacturers' protocol.

### 2.6 Animal and Experimental Design

Sixty male C57BL/6 mice (6–8 weeks old, 20 ± 2 g) were procured from the Hangzhou Laboratory Animal Center in Zhejiang Province, China. Prior to the experiment, the mice were housed and acclimatized for 1 week in a specific pathogen‐free environment where food and water are freely available. All experimental procedures followed strict compliance with ethics guidelines and were approved by the Institutional Animal Care and Use Committee of Fujian Medical University (IACUC FJMU 2022‐0025). The mice were randomly allocated to six experimental groups of 10 mice each. The negative control group (Group 1) consumed ordinary drinking water without DSS induction and received daily gavage of the vehicle (0.5% CMC‐Na in saline). The disease model control group (Group 2) received 2.5% DSS in drinking water for 7 days without drug treatment. Colitis was induced in Groups 3–6 by supplementing their drinking water with 2.5% DSS for 7 days, then a 2‐day recovery phase during which the animals were provided with regular drinking water. The positive control group (Group 3) was administered 300 mg/kg of 5‐ASA via intragastric gavage (Shen et al. 2022). MMN was administered via intragastric gavage once daily at doses of 25, 50, and 100 mg/kg in Groups 4–6, respectively, starting from day 1 (24 h before DSS induction) until day 9 (covering the entire 7‐day DSS exposure period and 2‐day recovery phase). Researchers blinded to group assignments conducted all outcome assessments following randomization. The animals were weighed, euthanized, and subsequently subjected to colon tissue dissection. Mice were randomized using a computer‐generated number sequence. To ensure objective assessment, investigators who were blinded to the group allocations performed all data collection, including DAI scoring, colon length measurement, histological evaluation, and biochemical analyses. The tissue samples were divided and processed using two distinct preservation methods: one portion was rapidly frozen and then preserved at −80°C for potential future applications, while the remaining sample was fixed with 4% formaldehyde solution and embedded in a paraffin wax block for subsequent experimental analyses.

### 2.7 Disease Activity Index (DAI)

In this study, DAI was scored by blinded investigators; weight loss, diarrhea, and bleeding were evaluated using a grading system derived from prior research (Jia et al. 2021). Weight loss was defined as the difference between primary and ultimate body weights. Diarrhea was characterized by the continuous passage of unformed or liquid fecal matter from the colon. Hematochezia was assessed by monitoring rectal bleeding in mice with diarrhea. The DAI was computed by summing the mean points for weight loss, fecal stickiness, and bleeding, followed by dividing the total by three. Following surgical resection, the colon was isolated from the proximal rectum and dissected toward the inferior pelvic region. Colonic length was calculated from the ileocolic intersectionto the proximal rectal margin.

### 2.8 Determination of Oxidative Stress and Antioxidant Enzymes

The concentrations of MDA and NO and the activity of SOD and GSH in colon tissue homogenates were quantified using commercially available kits through spectrophotometric analysis, following the standardized protocols by each manufacturer. Colonic tissue specimens were homogenized in lysis buffer using a mechanical homogenizer. The mixture was centrifuged for 10 min, and the supernatant was collected for analysis.

### 2.9 Measurement of Intracellular ROS

RAW 264.7 macrophages were seeded in six well plates with an initial concentration of 5 × 10^5^ cells/mL. After plating, the macrophages were pretreated with isolated MMN for 2 h, followed by a 22‐h incubation period with 2.5 μg/mL LPS. The cells were then treated with 10 μM of the fluorescent probe DCFH‐DA for ROS detection. Following incubation for 20 min in complete darkness at 37°C, the macrophages were washed thrice using serum‐free media. Afterwards, fluorescence signals were quantitatively assessed using a BD Biosciences FACS Vantage flow cytometer (San Jose, CA). FlowJo (Tree Star) was used to analyze the data.

### 2.10 Immunofluorescence Analysis

NF‐*κ*B/p65 transposition was assessed using immunofluorescence microscopy. RAW264.7 macrophages were incubated overnight in confocal dishes at 37°C and a 5% CO_2_ atmosphere. At the conclusion of the designated MMN incubation period, macrophages were treated with anti‐NF‐*κ*B p65 antibody (diluted 1:500) at 4°C for 12 h, and then with FITC‐bivalent antibody for 60 min at 37°C. After a 5‐min Hoechst staining step, nuclear images were captured using a Leica confocal imaging system.

### 2.11 Western Blotting

The influence of MMN on the expressions of p65, IKK*α*/*β*, I*κ*B*α*, Nrf2, p‐JAK2/p‐STAT3, and iNOS/eNOS proteins in macrophages and intestinal tissues of mice with UC was systematically evaluated by protein blot analysis. Total protein was isolated from mouse colon tissue using a dissolved buffer solution. Specifically, 30 mg of colonic tissue from each experimental group was homogenized in buffer to yield total protein extracts. Western blotting was performed according to a previously established protocol (Staal et al. 2011). Approximately 30 μg cell lysates were separated on an 8% SDS‐PAGE gel and subsequently transported to PVDF membranes using a Trans Blot Turbo Transfer System. The membrane was treated using the following primary antibodies: eNOS/iNOS, IKK*α*/*β*, p65, I*κ*B*α*, Nrf‐2, p‐STAT3, p‐JAK2, and *β*‐actin. Protein‐antibody interactions were detected and visualized with achemiluminescence detection system (Amersham Imager 600, USA).

### 2.12 Histological Evaluation

The entire colon was sliced lengthwise and rinsed thoroughly with cold phosphate‐buffered saline. The collected colon specimens were placed in 5% neutral‐cushioned formalin and fixed at room temperature for 24 h, then embedded in paraffin and sliced for subsequent histological analyses. After being fixed in paraffin and cut into 5 μm sections, the tissues were subjected to hematoxylin and eosin (H&E) staining for histological evaluation. The severity of colitis was assessed by two independent blinded observers using modified criteria based on the histological evaluation of H&E‐stained tissue sections. Images were captured using a Leica DM2000 LED light microscope (200× magnification, Leica Microsystems, Chicago, USA).

### 2.13 Immunofluorescence Staining

Mouse colon paraffin slices (5 μm thick) were degraphitized using dimethylbenzene, rehydrated using a graded alcohol series, and treated for an anti‐primary search. The cells were initially blocked using 10% normal goat serum for 1 h, followed by incubation with NF‐*κ*B primary antibodies (1:400 dilution) at 4°C overnight. Subsequently, fluorescent‐labeled secondary antibodies (1:500 dilution) were applied for staining. The nuclei were stained using DAPI solution and all subsequent procedures were performed under light‐protected conditions following secondary antibody incubation. Image acquisition was performed using an Olympus BX51 fluorescentmicroscope and subsequently processed/assessed using ImageJ.

### 2.14 Statistical Analyses

Data are presented as mean ± standard deviation (SD). Intergroup comparisons were conducted using the Student's *t*‐test for two groups, one‐way ANOVA (with Tukey's post hoc test) for multiple groups, and two‐way ANOVA (with Sidak's post hoc test) for datasets involving multiple factors. All statistical analyses were performed with SPSS 22.0 (IBM Corp., Armonk, NY, USA). A *p* value below 0.05 was considered statistically significant. Graphical representations were generated using GraphPad Prism 8 (GraphPad Software, San Diego, CA, USA) and Image J software. Significance levels are denoted as follows: ns (not significant, *p* ≥ 0.05), * (*p* < 0.05), and ** (*p* < 0.01).

## 3 Results

### 3.1 Impact of MMN on Cell Viability and NO Inhibition

As illustrated in Figure 1B, the viability of MMN‐treated macrophages remained consistently high (97.6%–100.4%) across the tested concentration range (0–20 μM), with no significant cytotoxicity observed. However, at 40 μM, cell viability decreased significantly to 66.2%. The results demonstrate that MMN has no cytotoxic effect on macrophages at concentrations below 20 μM. Excessive NO production serves as a critical biomarker of the inflammatory response in organisms and may induce vasodilation and microcirculatory dysfunction, thereby exacerbating inflammatory processes (Man et al. 2022). Herein, within this noncytotoxic concentration range (5–20 μM), MMN treatment exerted a notable dose‐responsive suppression of NO generation (Figure 1C). In summary, MMN was not cytotoxic at concentrations up to 20 μM and within this range, dose dependently inhibited NO synthesis.

### 3.2 Effect of MMN on Cytokine Generation in Macrophages

ELISA was used to detect the impact of MMN on IL‐6, IL‐1*β*, and TNF‐*α* secretion in LPS‐ stimulated macrophage culture supernatants. The concentrations of TNF‐*α*, IL‐1*β*, and IL‐6 were markedly increased in cell cultures incubated with LPS.

**FIGURE 2:**
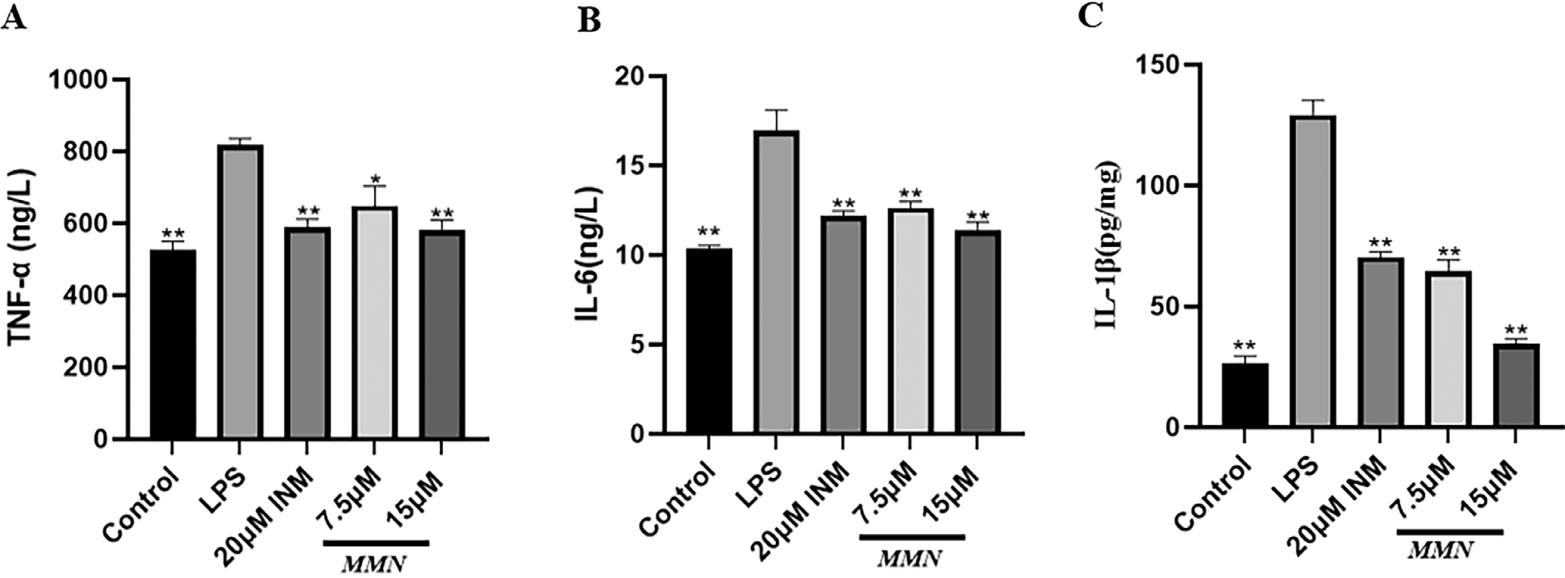
Impact of MMN on the secretion of TNF‐*α* (A), IL‐6 (B), and IL‐1*β* (C) in LPS‐activated macrophages. Pretreat macrophages with 7.5 and 15 μM MMN for 2 h before treating with 2.5 μg/mL LPS. After 22 h of incubation, the secretion of IL‐6, IL‐1*β*, and TNF‐*α* was detected with ELISA. The data are the mean ± SD (*n* = 3). Significance was assessed by one‐way analysis of variance using the Tukey test. **p < 0.01, *p < 0.05 relative to the LPS‐mediated group.

However, the rise was progressively attenuated with higher doses of MMN. MMN at concentrations between 5 and 20 μM markedly inhibited the release of inflammatory cytokines such as IL‐1*β*, IL‐6, and TNF‐*α*, as shown in Figure 2A–C. The findings show that MMN preconditioning significantly reduces LPS‐activated macrophage inflammation through dose‐dependently decreasing the production of TNF‐*α*, IL‐1*β*, and IL‐6.

### 3.3 Effects of MMN on ROS Production in Macrophages

**FIGURE 3:**
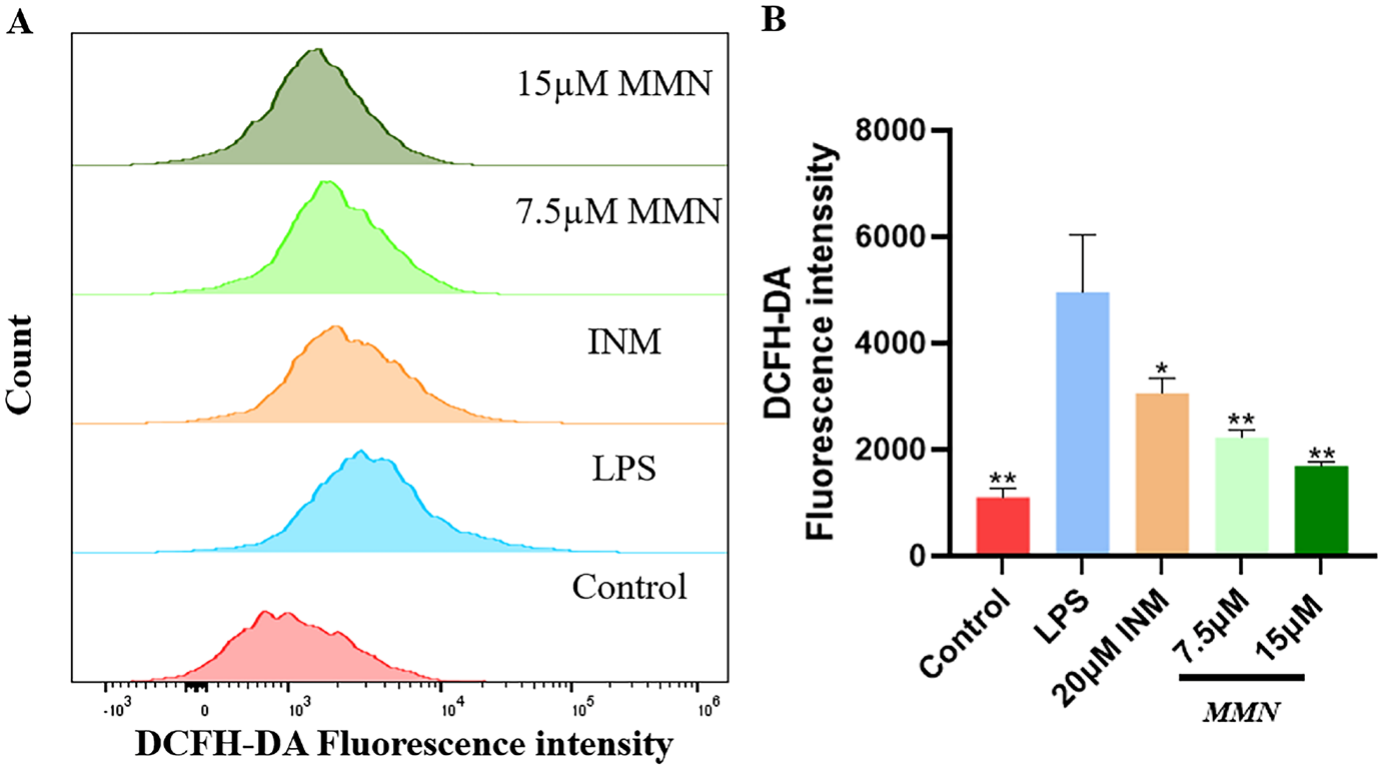
Impact of MMN on ROS generated by LPS stimulation. (A) Macrophages were preconditionedusing7.5, 15 μM MMN for 2 h prior to treatment with 2.5 μg/mL LPS for 22 h. Macrophages were cultured using 10 μM DCFH‐DA for 30 min. Macrophages were then harvested and immediately analyzed for DCF fluorescenceusing flow cytometry. The DCF fluorescence intensity was performed by randomly selecting the same number of cells. (B) The mean relative levels of ROS are displayedin a bar chart. The data is the mean ± SD (*n* = 3). Significance was assessed by one‐way ANOVA using Tukey test. Compared to the LPS‐mediated group, *p < 0.05, **p < 0.01.

The impact of MMN on ROS generation in macrophages activated by LPS was examined. ROS levels in macrophages significantly increased after 24 h of LPS treatment. MMN treatment significantly reduced this increase in a concentration‐ dependent fashion (Figure 3A,B), indicating that the anti‐inflammatory characteristics of MMN in RAW264.7 cells are linked to its antioxidant activity.

### 3.4 Exploration of the Role of MMN in NF‐κB Pathways

**FIGURE 4:**
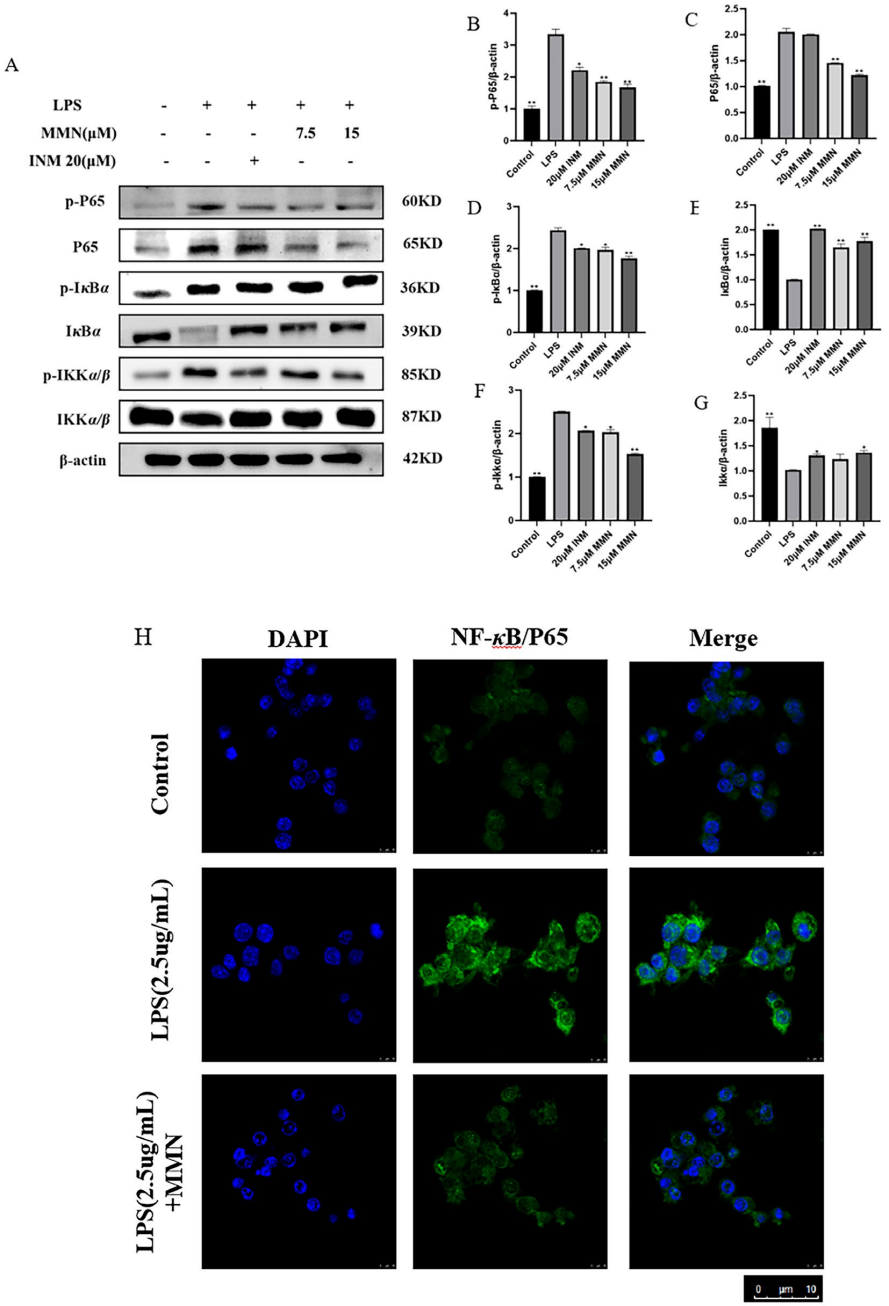
The NF‐*κ*B cascade is engaged in the anti‐inflammatory process for MMN. (A–G) Macrophages were preconditioned by MMN (7.5, 15 μM) for 2 h and then activated with LPS for 22 h. The expression of proteins P65, I*κ*B*α*, IKK*α*/*β* were determined via protein blotting. (H) Macrophages were preconditioned by MMN for 2 h, followed by LPS for 22 h. Translocation of p65 is measured via an immunofluorescence test as described in the Section 2. The data is the mean ± SD (*n* = 3). Significance was assessed by one‐way ANOVA using the Tukey test. Compared to the LPS‐mediated group, *p < 0.05, **p < 0.01.

Activation of NF‐*κ*B is key in regulating the expression of TNF‐*α*, iNOS, and other inflammation‐responsive genes (Singh et al. 2019). The NF‐*κ*B signaling cascade is essential in the progression of persistent inflammatory disorders. NF‐*κ*B is known to remain in an inactive state within the cytoplasm. In LPS‐mediated macrophages, NF‐*κ*B activation is critically dependent on IKK*α* and IKK*β*, which play essential roles in promoting I*κ*B phosphorylation and its subsequent degradation (Wu et al. 2020). The pivotal step in NF‐*κ*B initiation involves the phosphorylation of I*κ*Bs. Upon phosphorylation, I*κ*Bs undergo ubiquitination and are subsequently degraded at the 26*S* proteasome, leading to the liberation of NF‐*κ*B dimers. The liberated dimers subsequently migrate into the nucleus, where they initiate the expression of specific target genes (Liu et al. 2022). To investigate the activation status of the NF‐*κ*B signaling cascade, we examined the phosphorylation levels of key regulatory proteins, including p65, IKK*α*/*β*, and I*κ*Bα. As illustrated in Figure 4A–G, the protein levels of p65, IKK*α*/*β*, and I*κ*B*α* were significantly up‐regulated after LPS induction, indicating that LPS effectively activated the NF‐*κ*B signaling cascade. Treatment with MMN effectively counteracted the effects of LPS, demonstrating a dose‐dependent inhibition of p65, IKK*α*/*β*, and I*κ*B*α* phosphorylation. This finding indicates that MMN suppresses the transcription of inflammation‐responsive genes, which is associated with the inactivation of the NF‐*κ*B signaling cascade. Immunofluorescence staining analysis further demonstrated that MMN markedly inhibited the nuclear translocation of p65 (Figure 4H), confirming the critical involvement of NF‐*κ*B signaling in the anti‐inflammatory effects of MMN.

### 3.5 Effect of MMN on iNOS and JAK‐2 Expression in Macrophages

**FIGURE 5:**
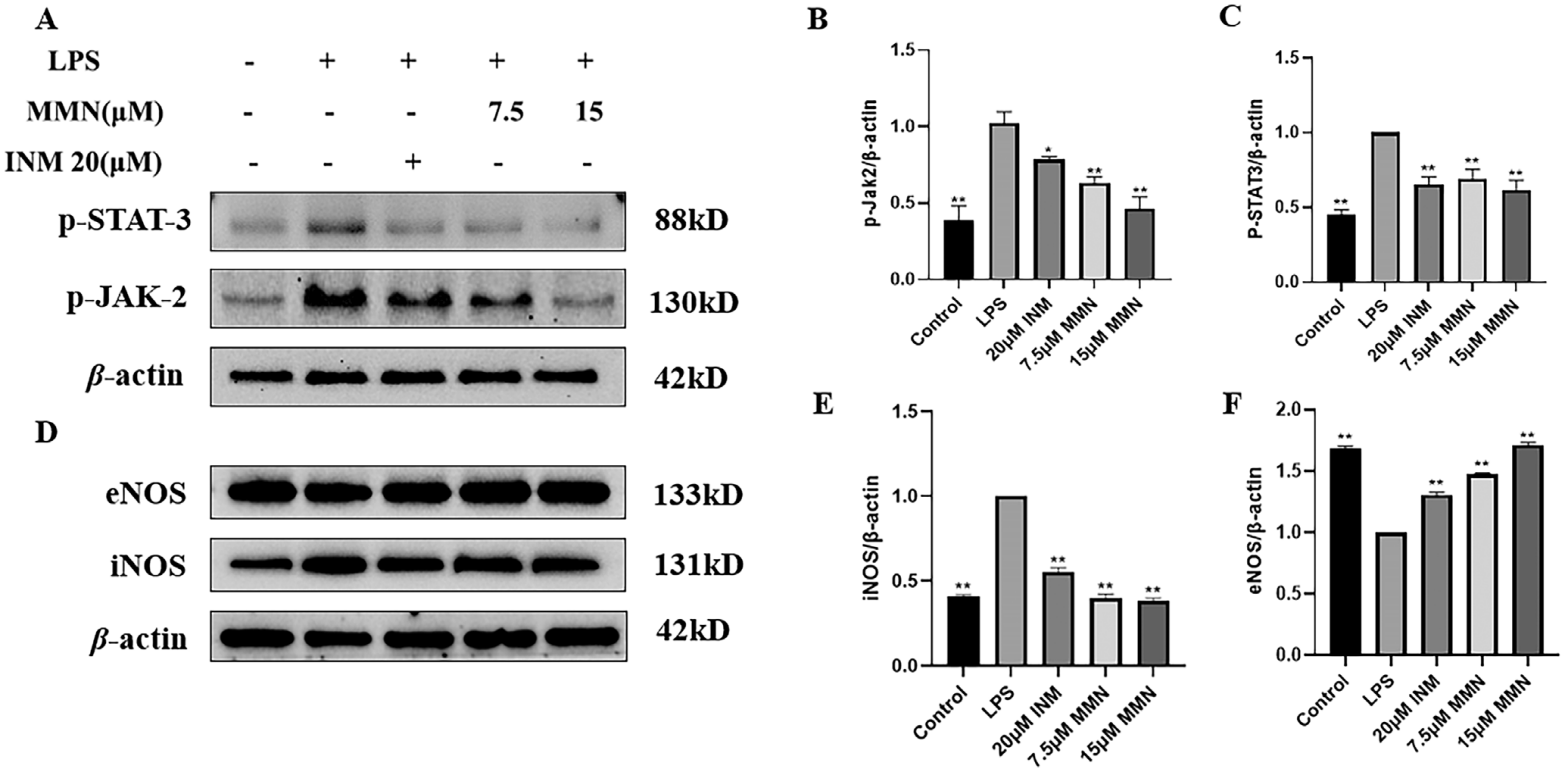
Impact of MMN on LPS‐stimulated *p‐*JAK2/*p‐*STAT3 and iNOS/eNOS protein expression. Pretreat macrophages with 7.5, 15 μM MMN for 2 h before treating with 2.5 μg/mL LPS. After 22 h of cultivation, (A–C), the levels of phosphorylated *p‐*JAK2 and *p‐*STAT3 were assayed by protein blotting. Band intensities for *p‐*JAK2 and *p‐*STAT3 were normalized to *β*‐Actin. iNOS and eNOS (D–F) protein levels were assayed by protein blotting. The data are the mean ± SD (*n* = 3). Significance was assessed by one‐way ANOVA using the Tukey test. *p < 0.05, **p < 0.01.

Our study examined how MMN influences inflammatory cytokine production in macrophages activated by LPS. Western blot analysis showed that, compared with LPS‐treated control cells, 15 μM MMN treatment significantly decreased the levels of phosphorylated p‐JAK2 and p‐STAT3 proteins (Figure 5A–C). As illustrated in Figure 5D–F, 15 μM MMN treatment significantly enhanced eNOS protein expression compared with the control, indicating upregulation of the eNOS pathway. In contrast, iNOS protein expression progressively decreased with increasing doses in the MMN‐administered group.

### 3.6 Effect of MMN on Clinical Symptoms of Colitis Caused by DSS

**FIGURE 6:**
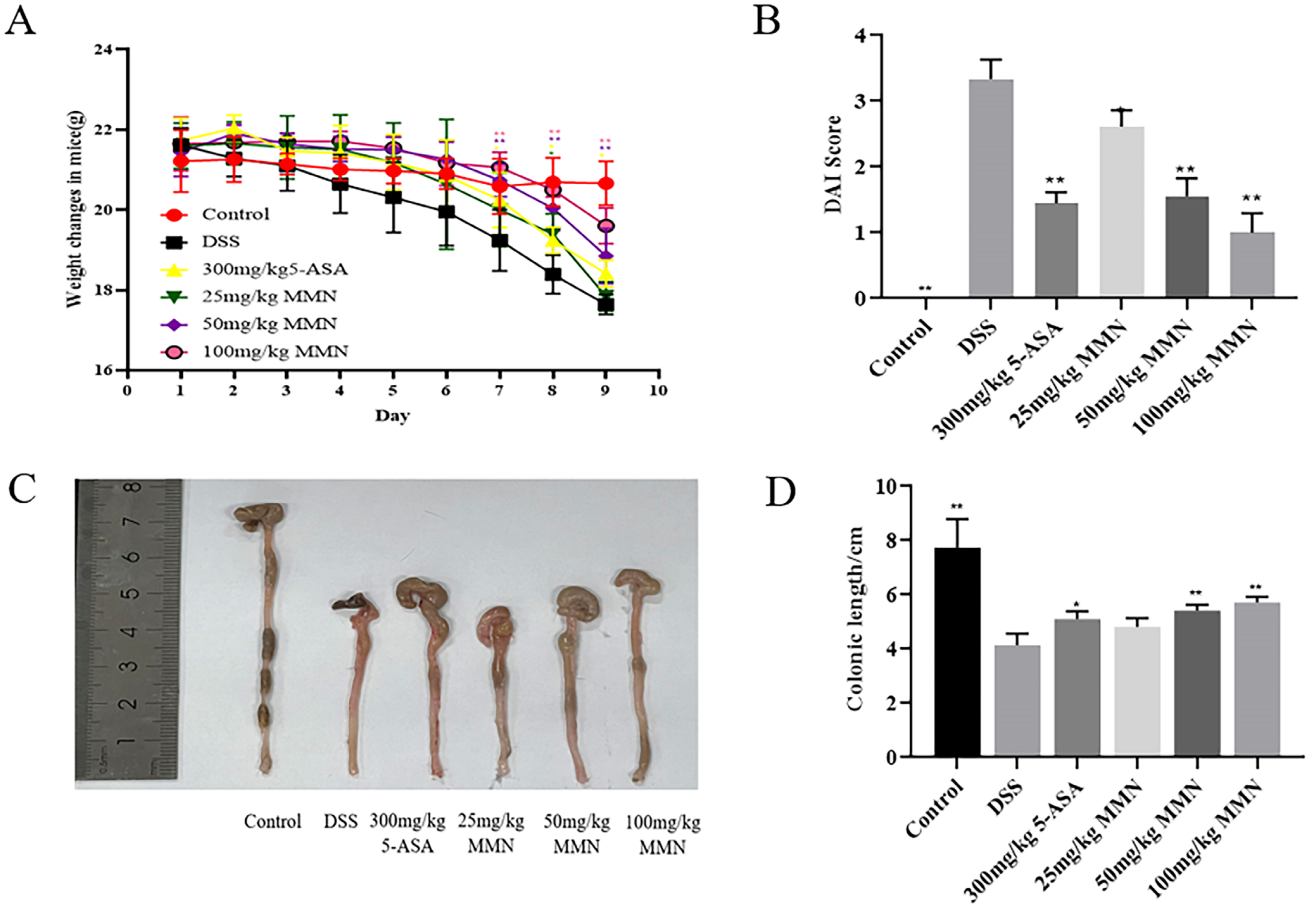
Effect of MMN on DSS‐stimulated UC in mice. MMN treatment notably reduces the clinical symptom of DSS‐stimulated colitis. For 7 days, mice received 2.5% DSS treatment, followed by 2 days of administration with either MMN (at doses of 25, 50, or 100 mg/kg) or no MMN. (A) Changes in body weight, (B) Disease severity score, Data for (A) and (B) are presented as mean ± SD (*n* = 10 in each group). Significance was analyzed using two‐factor analysis of variance and Sidak multiple comparison test. (C) Representative images of murine colons, (D) Length of the mouse colon. Data for (D) are presented as mean ± SD (*n* = 10 in each group). Significance was assessed by one‐way ANOVA followed by the Tukey multiple comparisons test. **p < 0.01 and *p < 0.05 relative to the DSS‐activated group.5‐ASA (5‐Aminosalicylic acid), as positive control.

Numerous investigations have demonstrated that DSS‐mediated models of experimental colitis, which are widely used to study the pathogenesis of IBD, exhibit multiple phenotypic characteristics closely resembling those observed in human UC (Salas et al. 2020). Within 9 days, mice with DSS‐induced colitis lost notably more weight than the healthy control animals; however, weight loss was markedly attenuated in the DSS plus MMN‐treated group (Figure 6A). Furthermore, the DAI scores, which encompassed weight loss, fecal thickness, and rectal bleeding, demonstrated a significant correlation with the observed weight loss (Figure 6B). Several studies have consistently demonstrated that DSS‐induced colitis is characterized by significant colonic tissue damage and a marked reduction in colon length (Pandey et al. 2017). As shown in Figure 6C,D, the colon length was markedly reduced in the UC group compared to controls, accompanied by marked congestion, severe edema, and notable tissue atrophy. The severity of these symptoms was significantly reduced in both the MMN and 5‐ASA treatment groups. These results clearly demonstrate that MMN administration effectively alleviates the clinical symptoms associated with DSS‐mediated acute colitis in a mouse model.

### 3.7 Effect of MMN on Inflammatory Response in Colitis

**FIGURE 7:**
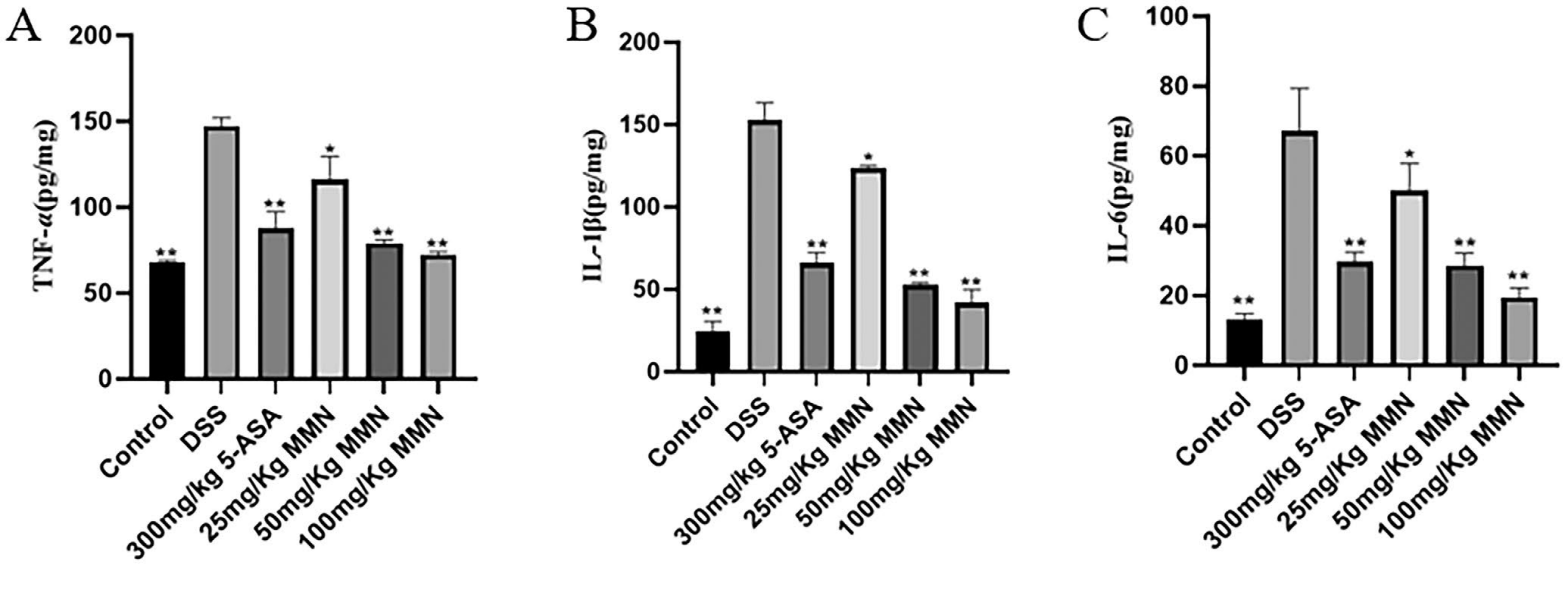
Impact of MMN on cytokine production in a DSS‐stimulated colitis model. (A) TNF‐α, (B) IL‐1β, and (C) IL‐6. Cytokine production was detected using an ELISA kit. Data are mean ± SD (*n* = 10 in each group). Significance was assessed by one‐way ANOVA using Tukey test. *p < 0.05 and **p < 0.01 relative to the DSS group.

The results shown in Figure 7 demonstrate markedly increased concentrations of pro‐inflammatory factors (IL‐6, IL‐1*β*, and TNF‐*α*) in colitis model mice relative to the control group (Figure 7A–C). Oral administration of MMN at doses of 50 and 100 mg/kg significantly suppressed the elevated levels of colitis‐related cytokines.

### 3.8 MMN Reduces Oxidative Stress

**FIGURE 8:**
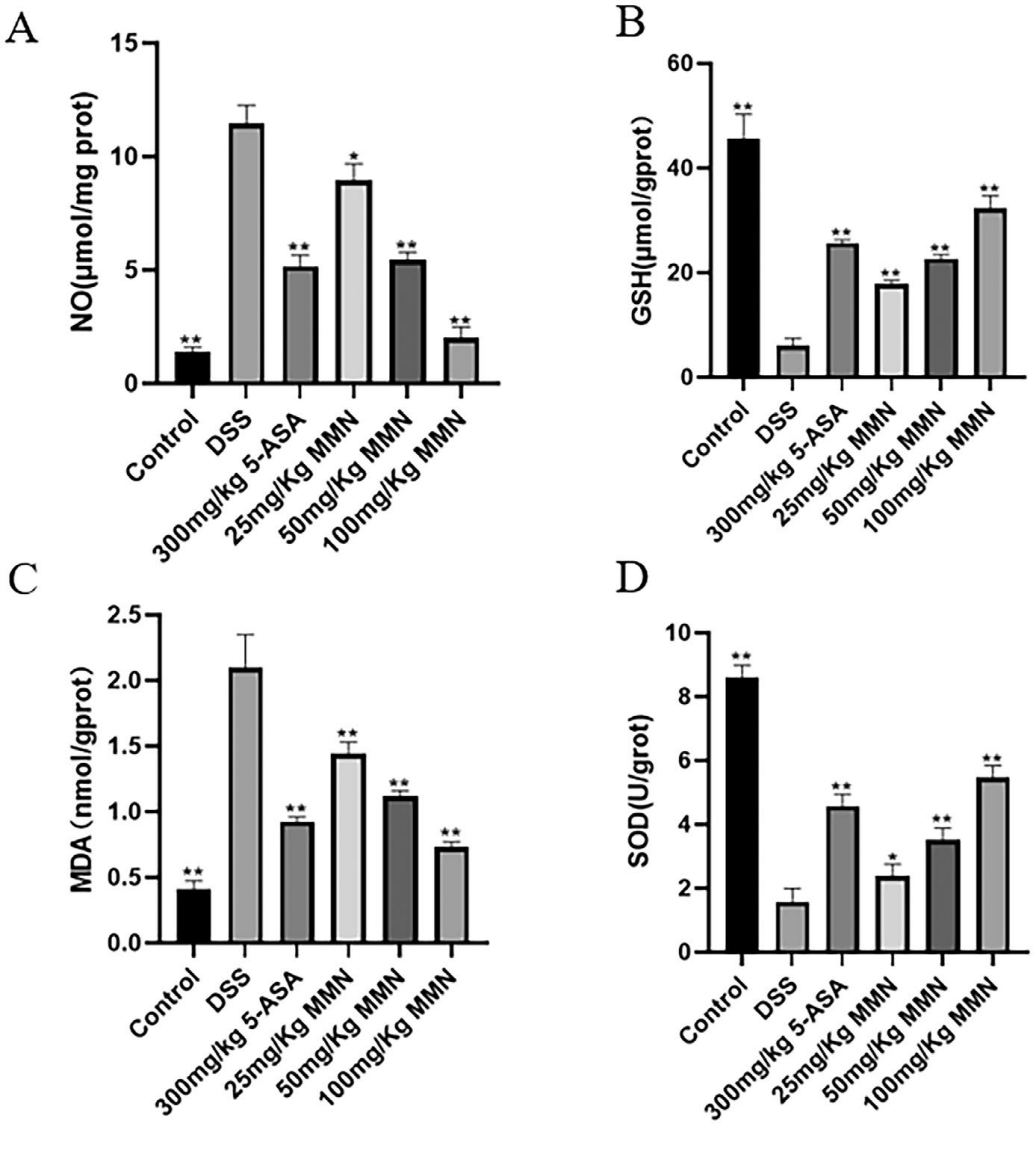
Antioxidant response of MMN in DSS‐stimulated UC. Level of NO (A), GSH (B), MDA (C), and SOD activity (D) in colonic homogenates. Data are mean ± SEM (*n* = 10 in each group). Significance was assessed by one‐way ANOVA using Tukey test. **p < 0.01 and *p < 0.05 relative to the DSS group.

As illustrated in Figure 8A–D, DSS treatment markedly elevated MDA and NO levels in mouse colonic tissues compared to the control, whereas MMN treatment significantly reduced MDA and NO concentrations relative to DSS treatment. SOD and GSH enzyme activities were markedly reduced in the DSS group compared to controls. Interestingly, MMN treatment up‐regulated SOD and GSH enzyme activities.

### 3.9 MMN Inhibits NF‐κB Signaling Cascades in Colonic Tissue

**FIGURE 9:**
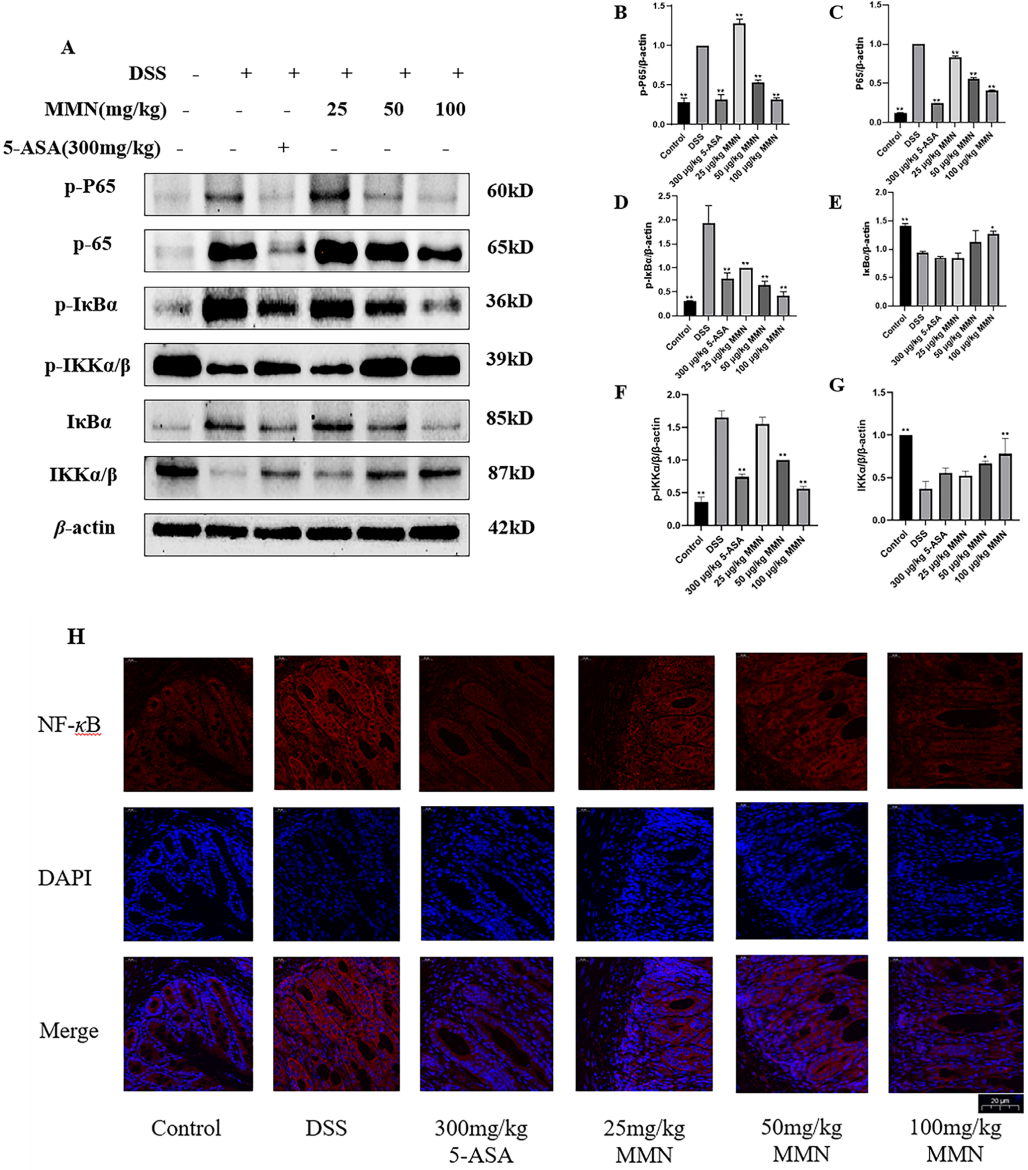
Inhibitory effects of MMN on NF‐*κ*B signaling cascade in UC mice. Western blot analyses for P65, IKK*α*/*β* and IκB*α* protein expression levels in colonic tissues (A–G). (H) Immunofluorescence analyses for P65 (red) in colonic mucosa (magnification 400×). DAPI is applied to nuclear staining (blue). Results show mean ± SEM (*n* = 3). Significance was assessed by one way ANOVA using Tukey test. *p < 0.05 and **p < 0.01 relative to DSS‐mediated group.

Transcription factors belonging to the NF‐*κ*B family serve as crucial modulators of gene expression and play critical roles in modulating both immune responses and inflammatory processes (You et al. 2017). Extensive experimental evidence has conclusively demonstrated that the NF‐*κ*B signaling cascade plays a central regulatory role in the pathogenesis and progression of IBD. Persistent activation of this cascade is commonly detected in inflamed colonic mucosa. To investigate the mechanism underlying MMN activity, the phosphorylation status of IKK*α*/*β*, p65, and I*κ*B*α* was assessed. The data in Figure 9A–G show a marked increase in phosphorylation levels in the DSS‐treated group compared to controls. These increases were significantly attenuated by DSS combined with MMN treatment at 50 and 100 mg/kg (Figure 9A–G). Immunofluorescence analysis revealed comparable expression patterns of NF‐*κ*B protein across all experimental conditions (Figure 9H). The experiment revealed that MMN markedly suppresses the NF‐*κ*B cascade in a UC mouse model, suggesting the involvement of this pathway in its protective effects.

### 3.10 Effect of MMN on Histological Changes in DSS‐Activated Colitis

**FIGURE 10:**
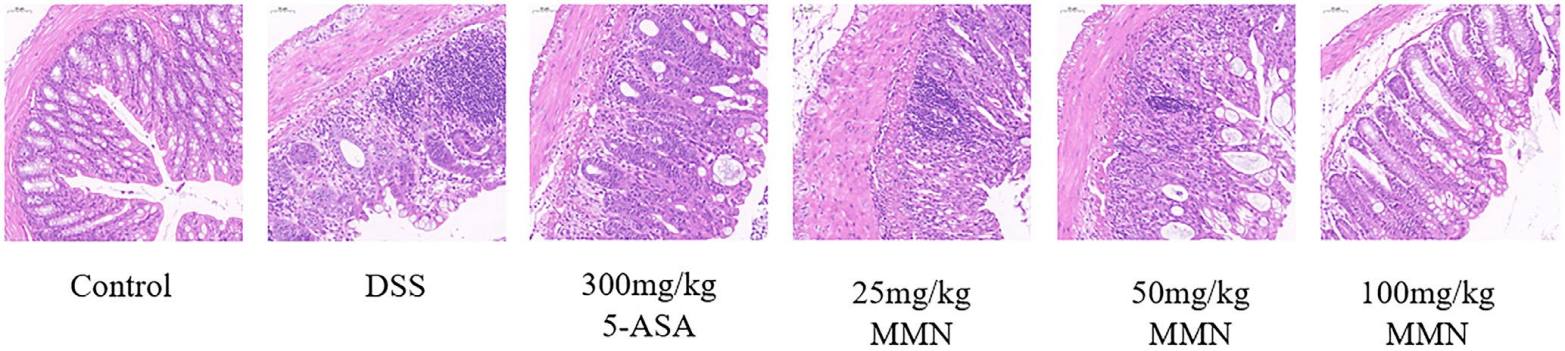
Protective influence of MMN on DSS‐activated mouse UC.H&E staining image in the colon at 200× magnification.

To evaluate the therapeutic efficacy of MMN on colitis‐associated clinical manifestations, a histological examination of colonic tissues was conducted, which revealed significant epithelial injury and inflammatory mast cell infiltration triggered by DSS treatment (You et al. 2017). As illustrated in Figure 10, histological analysis revealed distinct morphological differences between the experimental groups. The control group maintained an intact surface epithelium, well‐preserved colonic crypts, and normal interstitial and submucosal structures. In contrast, the DSS‐treated group exhibited severe pathological alterations, including distorted branchial crypts, prominent cryptitis, a substantial reduction in the number of crypts and cupped cells, extensive inflammatory cell infiltration, and marked submucosal edema. MMN administration markedly alleviated pathological alterations, including crypt architectural abnormalities, inflammatory cell infiltration, and tissue injury in the DSS‐mediated colitis model. Histological analysis demonstrated that MMN supplementation exerted protective benefits on DSS‐induced colitis in mice.

### 3.11 MMN Activates the Nrf‐2 Signaling Pathway in Mice With UC

**FIGURE 11:**
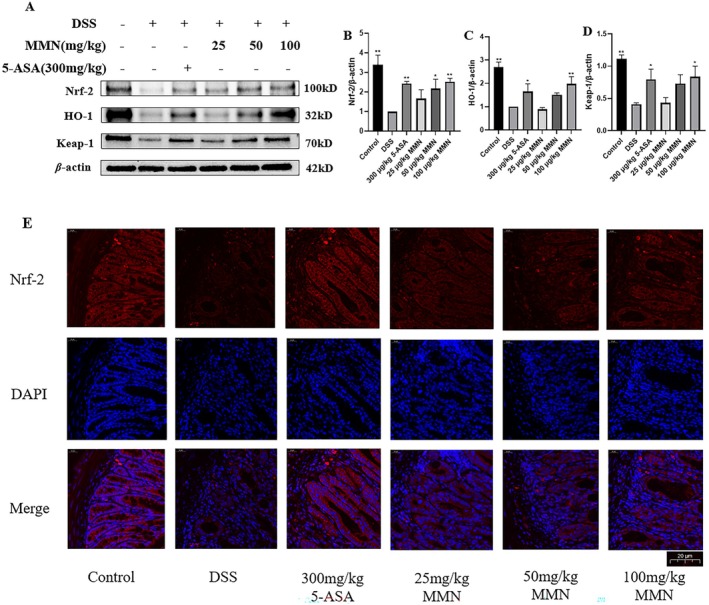
Regulation of the Nrf‐2 signaling pathway by MMN. (A, B) MMN treatment increased the levels of the crucial protein Nrf‐2. (C) HO‐1. (D) Keap‐1 participates in the Nrf‐2cascade in colonic tissues. (E) Immunofluorescence staining was performed to assess Nrf‐2 expression (red) in colon tissue sections at 400× magnification. DAPI is applied to stain nuclei (blue). Data are mean ± SEM (*n* = 3). Significance was assessed by one way ANOVA using Tukey test. *p < 0.05, **p < 0.01 relative to the DSS‐mediated group.

To explore the relationship between the anti‐inflammatory properties of MMN and the increased expression of HO‐1 and Keap1 through Nrf2 pathway activation, we conducted a detailed study. Nrf2 expression levels showed a clear concentration‐dependent increase following MMN administration; correspondingly, elevated expression of HO‐1 and Keap1 was also observed (Figure 11A–D). Immunofluorescence analysis of Nrf2 protein expression produced similar results (Figure 11E). These findings indicate that MMN exerts a strong modulatory effect on the Nrf2 pathway in a mouse model of UC, suggesting its potential contribution to the cytoprotective effects.

## 4 Discussion

The present study aimed to examine the suppressive effect of MMN on inflammatory responses. In vitro, its anti‐inflammatory effect was validated through significantly reducing LPS‐activated NO and key inflammatory factors, including IL‐6, TNF‐*α*, and IL‐1*β*, in macrophages. In vivo, MMN exhibited significant anti‐inflammatory activity in mice with DSS‐activated colitis, including attenuation of colonic shortening, reduction of DAI scores, decreased inflammatory cytokine levels, diminished inflammatory cell infiltration, and improvement of crypt damage.

The determination of administration doses was based on in vitro experimental data (including efficacy and toxicity), literature review, and pharmacokinetic calculations. In vitro experiments showed that the nontoxic concentration of the compound was 20 μM (10.14 μg/mL). The required target plasma concentration was 5–10 times this value (50.7–101.4 μg/mL). Considering the intragastric route of administration in this study, along with oral bioavailability (20%–50%) and the blood volume of mice (approximately 100 mL/kg), the actual dose was adjusted to 10–50 mg/kg. Accordingly, dose groups of 25, 50, and 100 mg/kg were established to cover the effective range and evaluate the safety of higher doses.

Extracts from the leaves, fruits, and bark of this *Melia azedarach* ‐derived triterpenoid compound can be ingested through diet or herbal preparations, and the daily exposure level in humans can be estimated based on traditional usage. According to the body surface area conversion formula (Human Equivalent Dose [mg/kg] = Animal dose [mg/kg] × [Animal Km value/Human Km value], with Km = 3 for mice and Km = 37 for humans), mouse doses of 25, 50, and 100 mg/kg correspond to human equivalent doses of approximately 2.03, 4.05, and 8.11 mg/kg, respectively, equivalent to approximately 122, 243, and 486 mg for a 60‐kg adult.

Even assuming that the compound content in fruits is lower than 0.01%, the intake from consuming 100 g of fruit would be less than 1 mg, and the daily intake from traditional decoctions (5–10 g of dried material) would be even lower. Thus, the human equivalent doses used in animal experiments (122–486 mg) are significantly higher than typical dietary intake. Accordingly, from a regulatory and safety perspective, if the compound is developed as a drug, a 28‐day repeated‐dose toxicity test is required. If developed as a dietary supplement, however, it must comply with Acceptable Daily Intake (ADI) standards.

The anti‐inflammatory properties of MMN were further supported by the reduced expression of inflammatory proteins in colonic tissues. MMN significantly improved pathological manifestations of colitis, including weight loss, diarrhea, hemostasis, and shortening of the colon, with therapeutic efficacy comparable to that of 5‐ASA.

Moreover, H&E staining demonstrated markedly elevated levels of inflammatory cell infiltration in the colonic mucosa of mice with colitis, which was attributed to the activation of chemokine signaling cascades and the subsequent recruitment of immune cells to inflammatory sites. This pathological manifestation was markedly ameliorated by MMN treatment. MMN administration significantly attenuated disease severity, as indicated by decreased DAI scores, and effectively promoted healing of colonic mucosal ulcers in mice with colitis. The experimental data clearly demonstrated that MMN significantly alleviated the inflammatory response in mice with UC.

To further evaluate the anti‐inflammatory properties of MMN, we assessed its impact on NO, TNF‐*α*, and ROS levels in LPS‐activated macrophages. NO and ROS are among the most important pro‐inflammatory mediators under inflammatory conditions. Numerous studies have shown that excessive NO production via iNOS is associated with various inflammatory diseases (Wu et al. 2007). Excessive ROS production induces oxidative stress by activating the NF‐*κ*B signaling cascade, representing an important molecular mechanism underlying inflammatory pathogenesis (Kim et al. 2012). Furthermore, the therapeutic efficacy of anti‐TNF‐*α* agents in IBD underscores the critical role of TNF‐*α* blockade in managing inflammatory conditions (Schmitz et al. 1999). The present study revealed that MMN suppressed ROS, TNF‐*α*, and NO generation in LPS‐ activated macrophages in a dose‐dependent manner.

IL‐6 and TNF‐*α* are critical pro‐inflammatory factors involved in the antitumor response, regulation of immune functions, and inflammatory processes (Lin et al. 2023). Among these, TNF‐*α* is released by activated macrophages, where it activates the NF‐*κ*B signaling cascade and modulates the secretion of various inflammation‐related cytokines (Cheon 2017). Activated macrophages that produce IL‐6 are crucial drivers of chronic infections and autoimmune disorders (Akbari and Hassan‐Zadeh 2018). Therefore, modulating the levels of pro‐inflammatory factors represents a promising therapeutic strategy for attenuating excessive inflammatory responses.

In this study, macrophage levels of IL‐6 and TNF‐*α* were notably elevated in the model group compared with controls (Figure 2). This increase is primarily attributed to LPS activation, which triggers robust production of pro‐inflammatory cytokines in cellular systems (Zhou and Zhu 2009). MMN (7.5 and 15 μM) significantly attenuated LPS‐induced effects, with TNF‐*α* levels showing a marked reduction compared with the model group, reaching levels comparable to those of the control group (Figure 2A). Similarly, IL‐6 production was significantly decreased.

Our experimental results demonstrated that MMN significantly suppressed LPS‐induced transcription of pro‐inflammatory factors in macrophages. The NF‐*κ*B signaling cascade serves as a pivotal regulatory hub in modulating the inflammatory response (Tak and Firestein 2001). In unstimulated cells, NF‐*κ*B normally remains inactive by binding to I*κ*Bs, which sequester transcription factors in the cytoplasm. Following the degradation of I*κ*Bs, the liberated NF‐*κ*B dimers are translocated into the nucleus where they initiate transcription of the targeted gene (Brown et al. 1995). In LPS‐stimulated macrophages, MMN dose‐dependently inhibited I*κ*B*α* phosphorylation (Figure 4), indicating its potential to suppress NF‐*κ*B signaling cascade activation. The phosphorylation of I*κ*B*α* is regulated by a cascade of kinases, primarily involving I‐*κ*B kinases (Mercurio et al. 1997). MMN may exert its inhibitory effect by targeting IKK, thereby suppressing I*κ*B*α* phosphorylation. The direct kinase targets of MMN and their potential regulatory effects on additional signaling pathways warrant further investigation. Collectively, these findings suggest that MMN suppresses the inflammatory response of LPS‐activated macrophages, an effect that is associated with the inactivation of the NF‐*κ*B signaling cascade, supporting its potential anti‐inflammatory effects.

Oxidative damage is acknowledged as an important factor in the pathogenesis of inflammatory disorders (Qian et al. 2020). Prior research has demonstrated that SOD and GSH‐Px play crucial roles as antioxidants in various biological systems. SOD catalyzes the conversion of superoxide anions to hydrogen peroxide, thereby eliminating free radicals and preserving oxidative homeostasis within the body. In addition, GSH plays an essential role in protecting cell membranes from oxidative injury through its catalytic function in the reduction of hydrogen peroxide to water. These two components function synergistically to regulate and preserve oxidative equilibrium within an organism (Chen et al. 2023).

To examine the impact of MMN on antioxidant enzyme activity, the levels of GSH and SOD were quantitatively assessed in murine colonic tissues. The DSS‐exposed group showed a notable reduction in GSH and SOD activity in colonic tissues. Administration of MMN effectively attenuated these DSS‐induced detrimental effects and mitigated the reduction in SOD and GSH activity. MDA is the major product of lipid peroxidation and is a reliable biomarker for evaluating the degree of oxidative injury to lipids in biological systems (Ezhilarasan 2018). In this study, administration of DSS significantly elevated MDA levels relative to those in the control group (Figure 8). Nevertheless, MDA content was significantly reduced following MMN treatment, suggesting enhanced antioxidant enzyme activity and reduced lipid peroxidation. In summary, MMN exhibited a protective role in DSS‐activated colon oxidative damage in mice by restoring the activity of antioxidative enzymes, thereby reducing MDA production and alleviating inflammatory injury.

As a pivotal inflammatory mediator, NO is an established marker of inflammation in animal models and modulates inflammatory responses via iNOS (Jayawardena et al. 2020). In the DSS‐activated group, NO levels were significantly increased after DSS treatment, whereas colonic NO concentrations were markedly reduced following MMN treatment (Figure 8A), suggesting that MMN plays an anti‐inflammatory role through inhibiting NO production.

Cytokines, a diverse group of endogenous signaling peptides, are primarily synthesized and secreted by immune cells and exert potent biological effects through their crucial role in mediating both immune regulation and inflammatory responses (Lin et al. 2023). Following DSS administration, innate immune responses are triggered, inducing an inflammatory cascade characterized by substantial up‐regulation of pro‐inflammatory cytokines (Moon et al. 2019). Our investigation of the effect of MMN on pro‐inflammatory factor levels demonstrated that IL‐6, TNF‐*α*, and IL‐1*β* concentrations were notably increased in the DSS‐activated group compared to controls. Notably, DSS stimulation triggers the secretion of pro‐inflammatory factors, enhancing inflammatory reactions in mice (Shen et al. 2022). In contrast, MMN intervention notably decreased IL‐6, IL‐1*β*, and TNF‐*α* levels. These findings provide further evidence that MMN supplementation effectively attenuates DSS‐induced acute inflammatory injury in murine models by significantly reducing pro‐inflammatory cytokine levels in colonic tissue.

Heme oxygenase1 (HO‐1) and Kelch‐like ECH‐associated protein 1 (Keap1) facilitate the degradation of heme, producing bilirubin, free iron, and carbon monoxide. Studies have shown that bilirubin possesses significant antioxidant properties (Ahmed et al. 2017). Recent studies have demonstrated that diverse triterpenoids derived from medicinal plants can upregulate the expression of HO‐1 and Keap1, thereby enhancing cellular antioxidant capacity and suppressing inflammatory responses through activation of the Nrf2/ARE signaling cascade. Preemptive overexpression of HO‐1 and Keap1 has been demonstrated to markedly reduce the production of inflammatory factors, particularly NO and IL‐6, upon subsequent inflammatory challenges (Drummond et al. 2019). Furthermore, marked inflammatory responses were observed in HO‐1 and Keap1 knockout murine models. These findings indicate that HO‐1 and Keap1 may serve as promising molecular targets for anti‐inflammatory therapeutic strategies (Paine et al. 2010). Nrf‐2, a key transcription factor activated by oxidative stress, regulates the expression of HO‐1 and Keap1 by translocating them to the nucleus and inducing the transcription of these phase 2 detoxifying enzymes as part of the antioxidant response. Nrf‐2‐driven upregulation of phase II detoxifying enzymes represents a critical antioxidant defense mechanism that provides cytoprotection through efficient detoxification and neutralization of ROS and xenobiotics (Zhou et al. 2019). In our study, MMN treatment in mice with UC induced Nrf2 upregulation, which was followed by elevated HO‐1 and Keap1 expression, suggesting that its anti‐inflammatory effects may be mediated, at least in part, through the activation of HO‐1 and Keap1.

In this study, we systematically investigated the modulation of NF‐*κ*B activation by MMN using an in vivo model of IBD. Our findings demonstrated that MMN significantly ameliorated DSS‐induced colitis through a dual mechanism of attenuating colon tissue injury and suppressing the expression of key inflammatory mediators. Furthermore, our findings demonstrated that MMN significantly attenuated DSS‐mediated colon shortening and modulated the levels of key inflammatory mediators, particularly IL‐6 and TNF‐*α*, in colonic tissue. In the DSS‐activated colitis model, MMN administration consistently attenuated inflammatory responses, as reflected by decreased concentrations of pro‐inflammatory mediators, and improved bowel mucosal integrity, as demonstrated by better histological scores.

Furthermore, while our data associate MMN's anti‐inflammatory effects with NF‐*κ*B inhibition, future studies using specific pathway inhibitors or genetic approaches are needed to establish causality. Similarly, the functional necessity of the Nrf2/HO‐1/Keap1 axis in the protective effects of MMN was not definitively established. Although our data show pathway activation, future studies utilizing specific pharmacological inhibitors, such as the Nrf2 inhibitor ML385 or the HO‐1 inhibitor SnPP, are required to confirm the causal involvement of this signaling cascade.

While preliminary acute toxicity and short‐term studies have established the basic safety profile of MMN, several limitations must be acknowledged to facilitate its clinical translation. Most notably, the current findings require validation using human primary cells to better predict clinical responses. Additionally, comprehensive pharmacokinetic characterization remains to be established. Addressing these key aspects—particularly through human cell‐based verification and systematic pharmacokinetic profiling—would significantly enhance the translational potential of MMN as a therapeutic agent.

Current research indicates that MMN may help alleviate intestinal inflammation in mice with colitis. Theoretically, these results indicate that natural products derived from medicinal plants can serve as a valuable source of healthcare drugs, offering further insight into the significance of traditional medicines, such as *Melia azedarach* . It is of critical importance to explore novel perspectives for traditional herbs and plant‐derived compounds utilized in the treatment of various diseases. Due to the limitations associated with many natural products—such as adverse effects, including hepatotoxicity, high‐throughput screening and molecular docking analysis are commonly employed to identify target proteins or enzymes involved in their mechanisms of action. Nevertheless, further in‐depth investigation is necessary to elucidate the pharmacodynamics of different phytochemicals and to establish their clinical efficacy.

## 5 Conclusions

Herein, we systematically evaluated the anti‐inflammatory activity of MMN by modeling LPS‐activated inflammatory damage in macrophages. The findings demonstrated that MMN treatment significantly attenuated the production of pro‐inflammatory factors, notably reducing TNF‐*α* and IL‐6 levels in macrophages. In addition, treatment enhanced intracellular antioxidant enzyme activity, ameliorated oxidative damage, and protected cells against LPS‐induced inflammatory responses. In vivo, MMN decreased pro‐inflammatory cytokine levels in mouse colons, improved blood indices, and preserved organ morphology. It also alleviated DSS‐mediated oxidative damage in colonic tissue, thereby safeguarding mice from acute DSS‐induced inflammation. Overall, the anti‐inflammatory efficacy of MMN and its protective effects against DSS‐induced acute inflammation were systematically demonstrated using in vivo and in vitro experimental approaches. These results provide a solid theoretical foundation for further research into the potential of MMN as a therapeutic option in treating severe inflammatory conditions.

## Author Contributions

**Fan Cao:** conceptualization (equal); methodology (equal); writing – original draft (equal); investigation (equal); formal analysis (equal). **Hua Wu:** methodology (equal); investigation (equal); formal analysis (equal). **Zhen He:** investigation (equal); data curation (equal). **Mengxin Di:** investigation (equal); data curation (equal). **Bin Liu:** investigation (equal); data curation (equal). **Zhenwei Chen:** investigation (equal); data curation (equal). **Jieming Xie:** formal analysis (equal); resources (equal); visualization (equal). **Yonghong Zhang:** conceptualization (equal); resources (equal); writing – original draft (equal); supervision (equal). **Xinhua Ma:** validation (equal); formal analysis (equal); resources (equal); visualization (equal); supervision (equal).

## Funding

This study was supported by Fujian Provincial Natural Science Foundation of China (2020J01619, 2023J01550) and Open Project Foundation of Fujian Key Laboratory of Natural Medicine Pharmacology (FJNMP202202).

## Ethics Statement

The study protocol was approved by the Ethics Commission of Fujian Medical University (IACUC FJMU 2022‐0025).

## Conflicts of Interest

The authors declare no conflicts of interest.

## Supporting information


**Figure S1:** fsn371310‐sup‐0001‐supinfo.docx. ^1^H‐NMR spectrum (600 MHz) of MMN in CDCl_3_.
**Figure S2:** ^13^C‐NMR spectrum (150 MHz) of MMN in CDCl_3_.
**Figure S3:** HR‐ESI‐MS spectrum of MMN.
**Figure S4:** HPLC spectrum of MMN.

## Data Availability

The data supporting the conclusions of this article are included in the manuscript.
