# Supplementary material for: Anti‐Inflammatory Effect of 21α‐Methylmelianol In Vitro and In Vivo via NF‐κ B/STAT Signaling Pathway Modulation
Source: Food Sci Nutr. 2025 Dec 4;13(12):e71310. doi: 10.1002/fsn3.71310 (PMC12678060; doi:10.1002/fsn3.71310)
Supplement: Supplementary file 1 — Figure S1: fsn371310‐sup‐0001‐supinfo.docx. 1H‐NMR spectrum (600 MHz) of MMN in CDCl3. Figure S2: 13C‐NMR spectrum (150 MHz) of MMN in CDCl3. Figure S3: HR‐ESI‐MS spectrum of MMN. Figure S4: HPLC spectrum of MMN. [file FSN3-13-e71310-s001.docx]

**Anti-inflammatory effect of 21α- methylmelianol in vitro and in vivo via NF-κB/STAT signaling pathway modulation**

**List of Supporting Information**

**Figure S1**:^1^H-NMR spectrum (600 MHz) of MMN in CDCl_3_.

**Figure S2**:^13^C-NMR spectrum (150 MHz) of MMN in CDCl_3_.

**Figure S3**: HR-ESI-MS spectrum of MMN

**Figure S4:** HPLC spectrum of MMN

**Suppl 1.** The Table of Animal Experimental Ethical Inspection (IACUC FJMU 2022-0025)


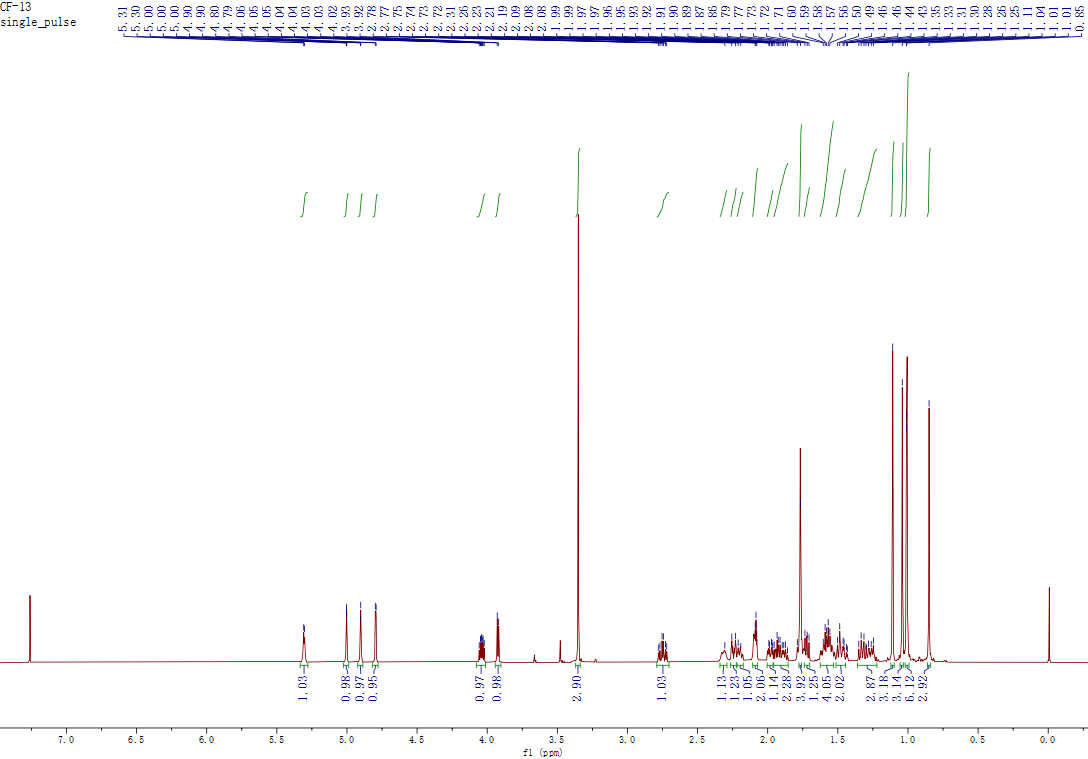


Figure S1:^1^H-NMR spectrum of MMN (600 MHz, chloroform-*d*)


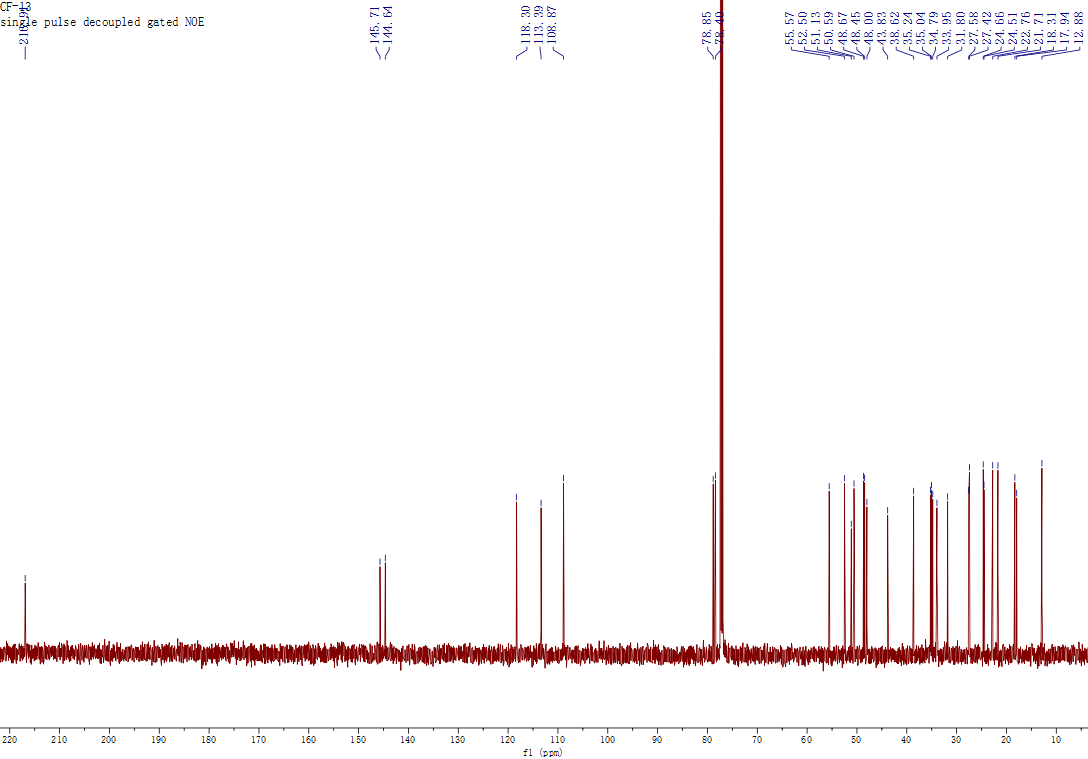


Figure S2:^13^C-NMR spectrum of MMN (150 MHz, chloroform-*d*)


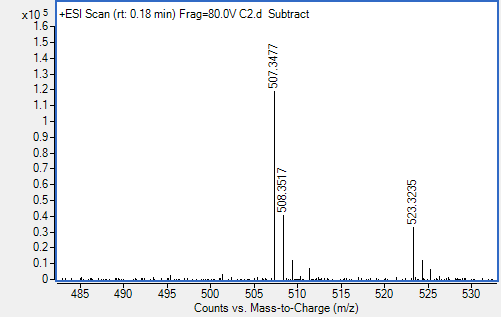


Figure S3:HR-ESI-MS spectrum of MMN

Figure S4: HPLC spectrum of MMN


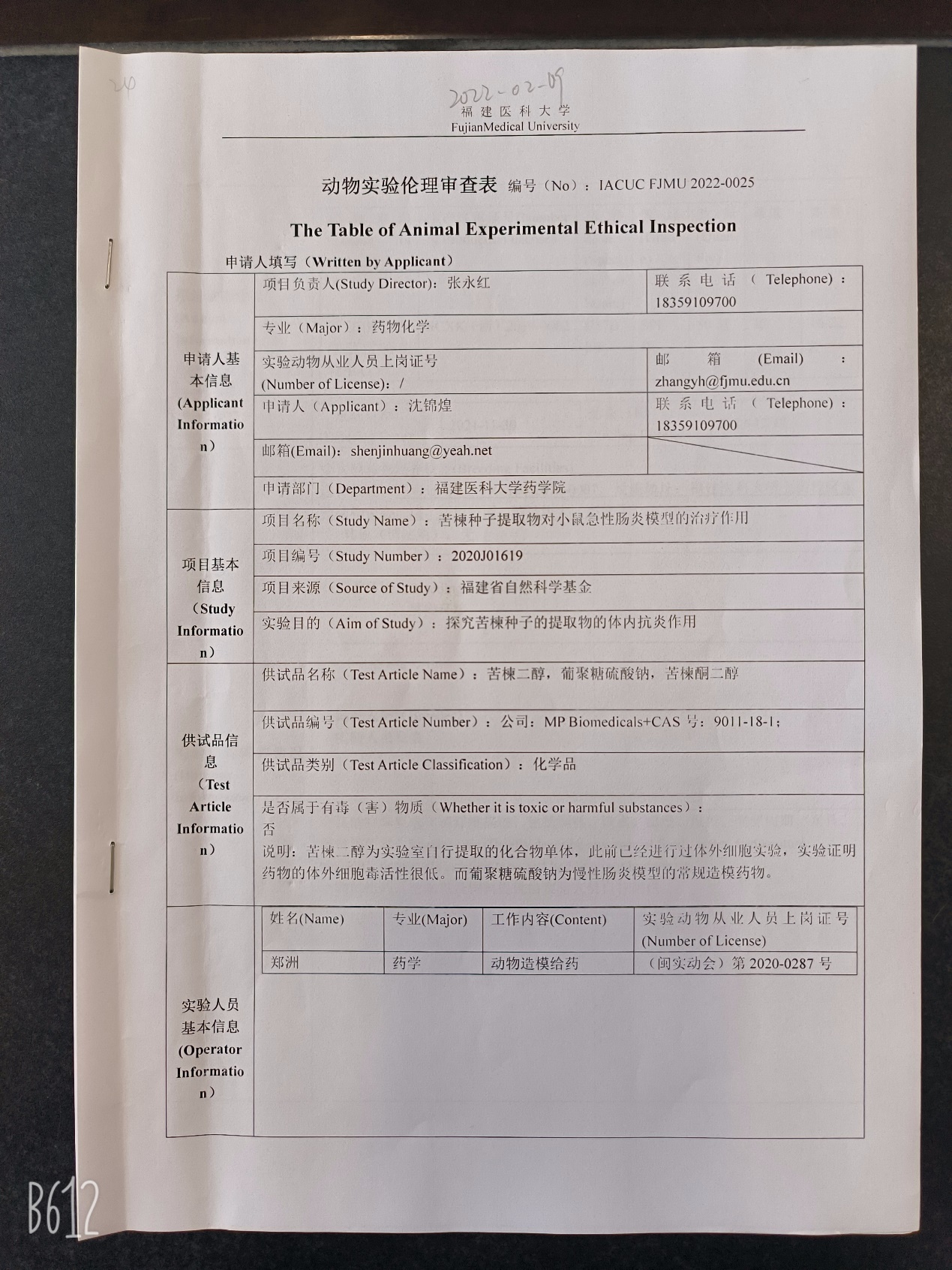


**IACUC-1**


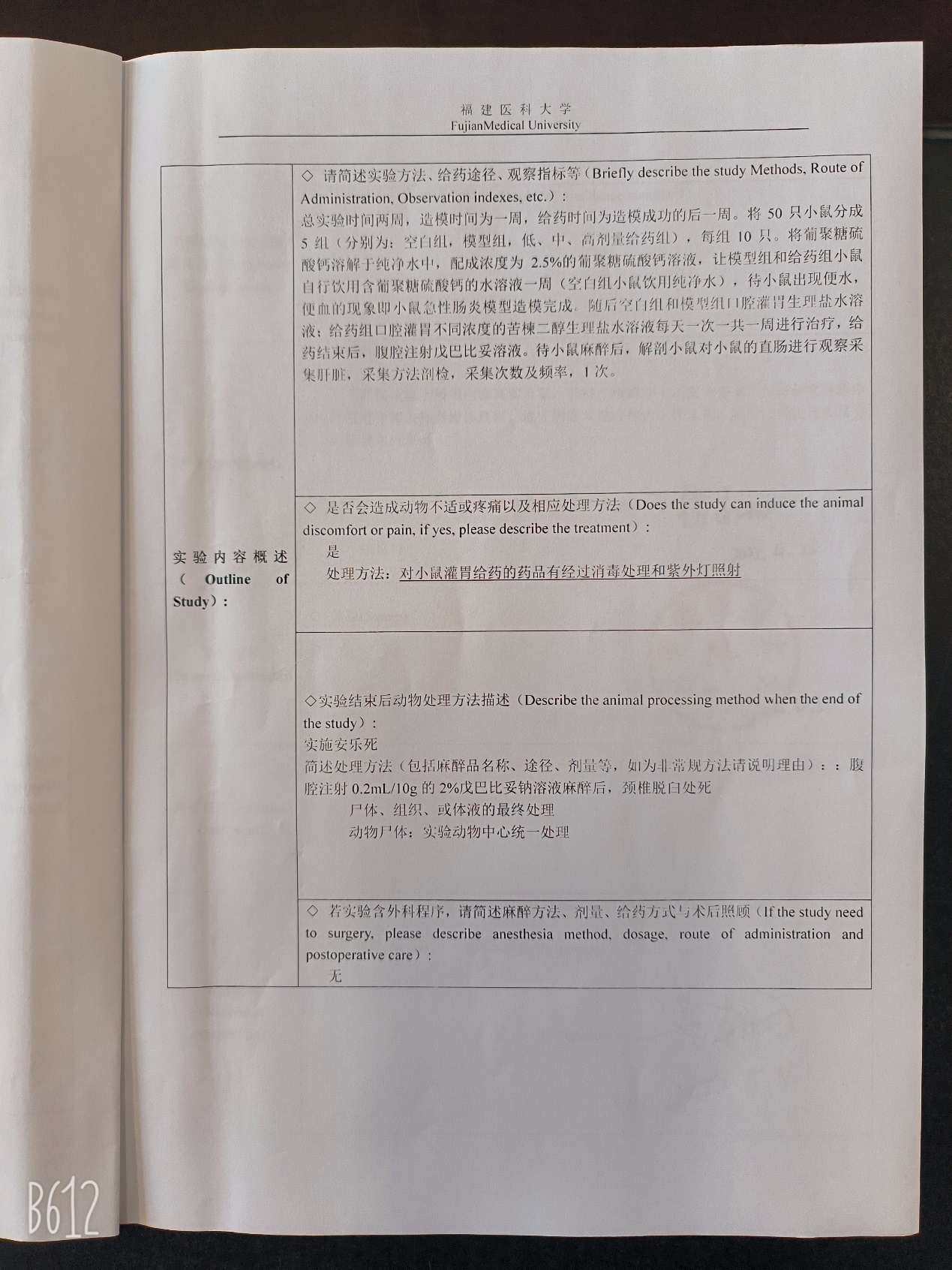


**IACUC-2**


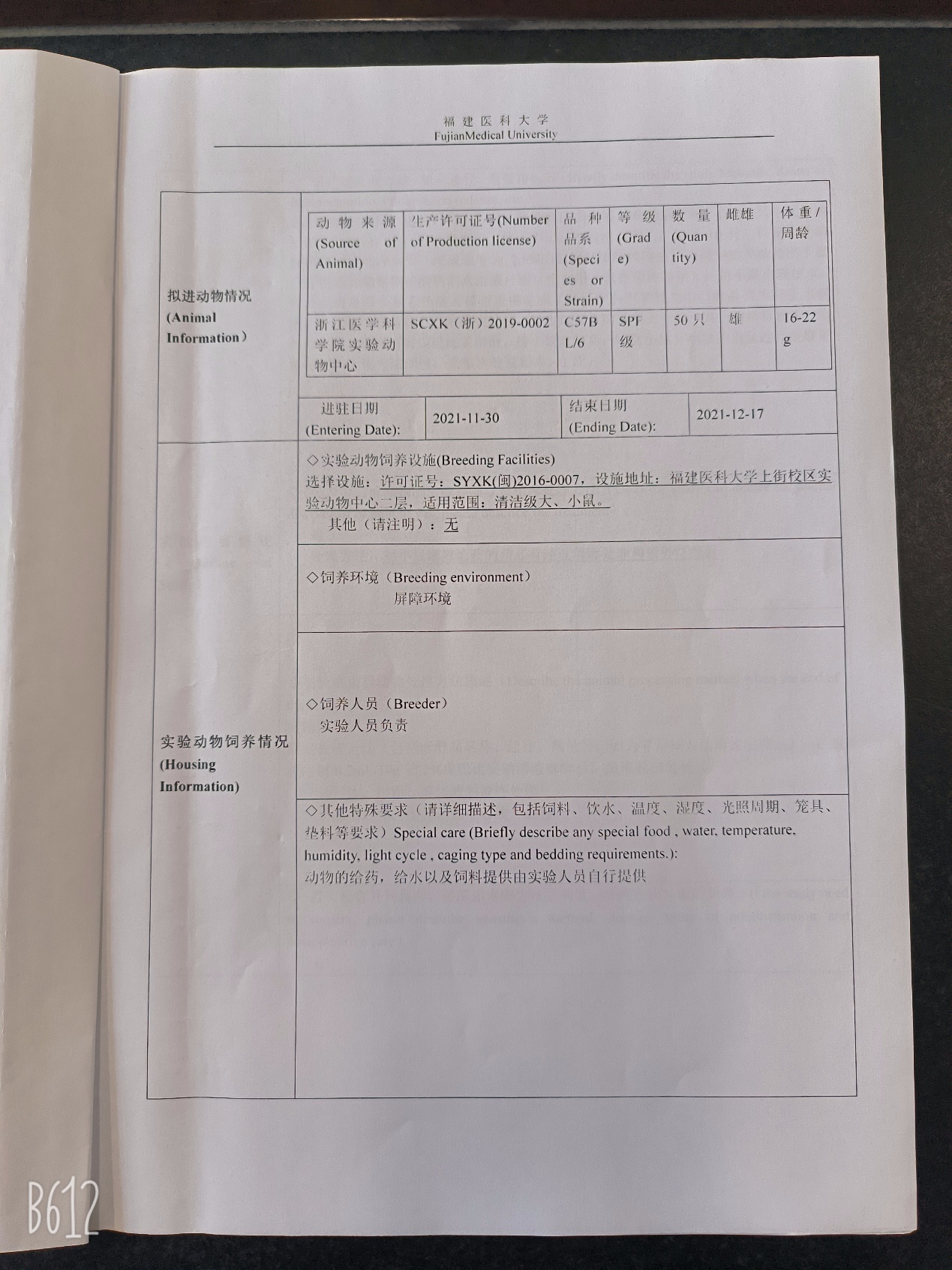


**IACUC-3**


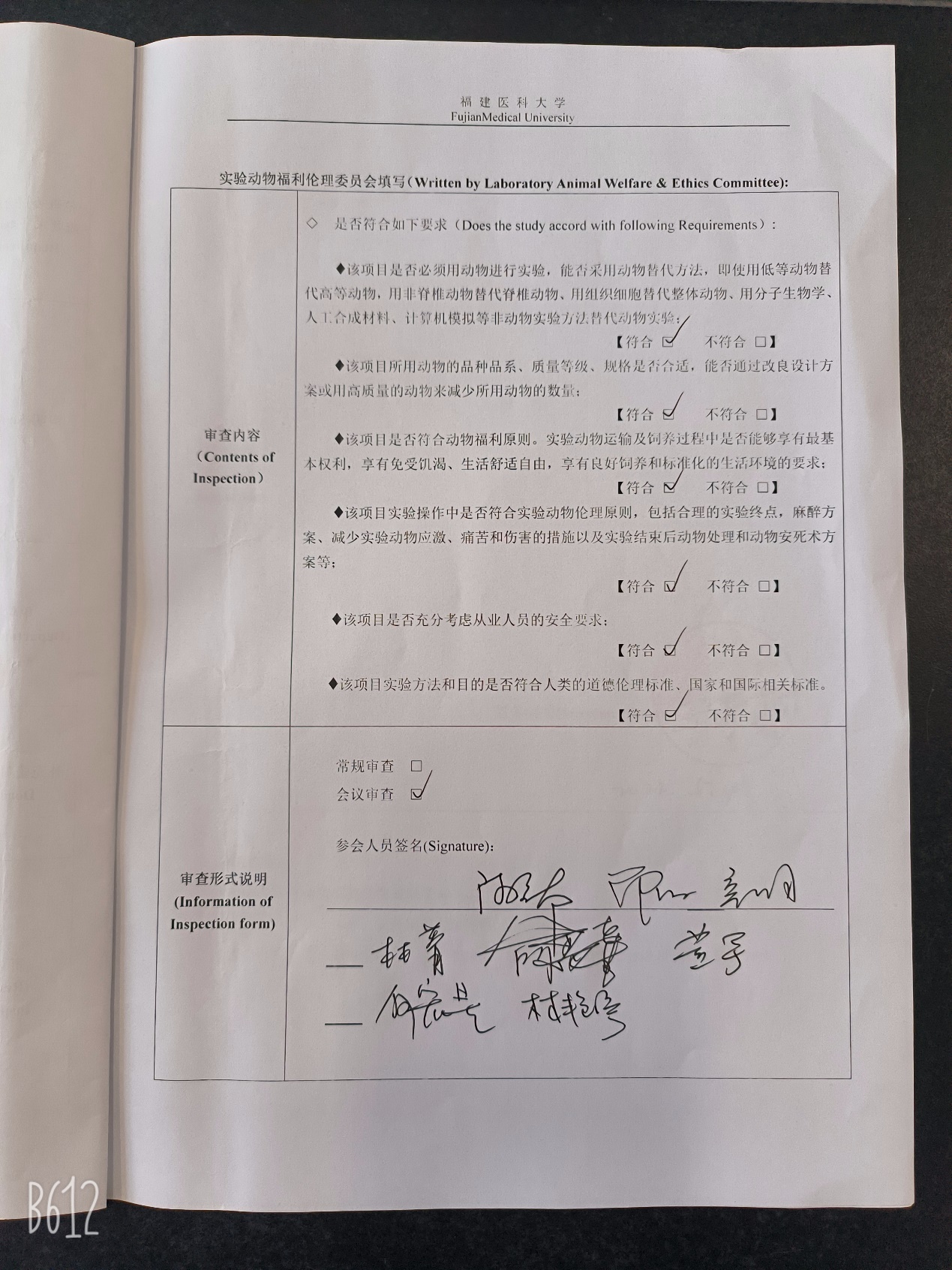


**IACUC-4**


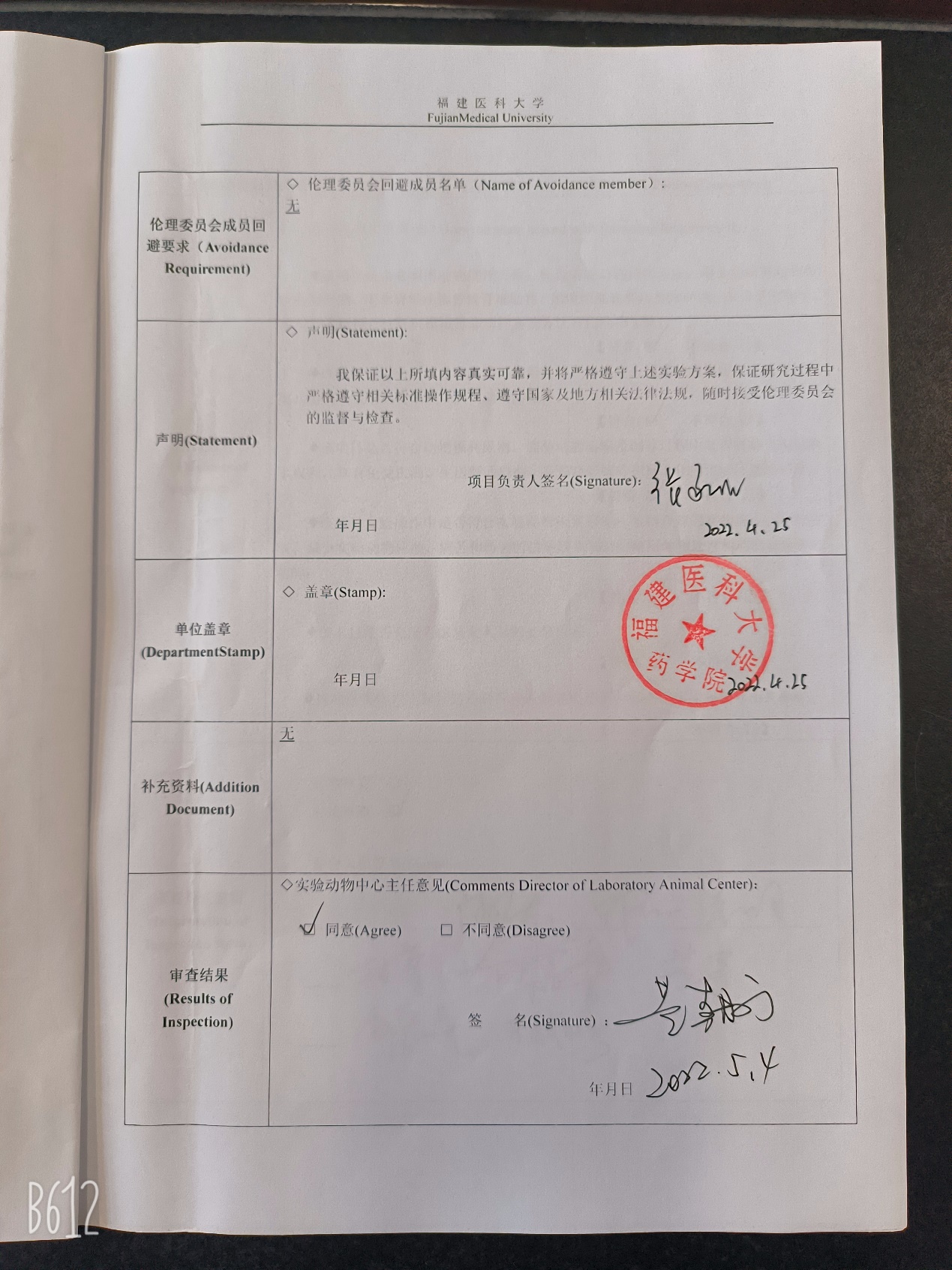


**IACUC-5**


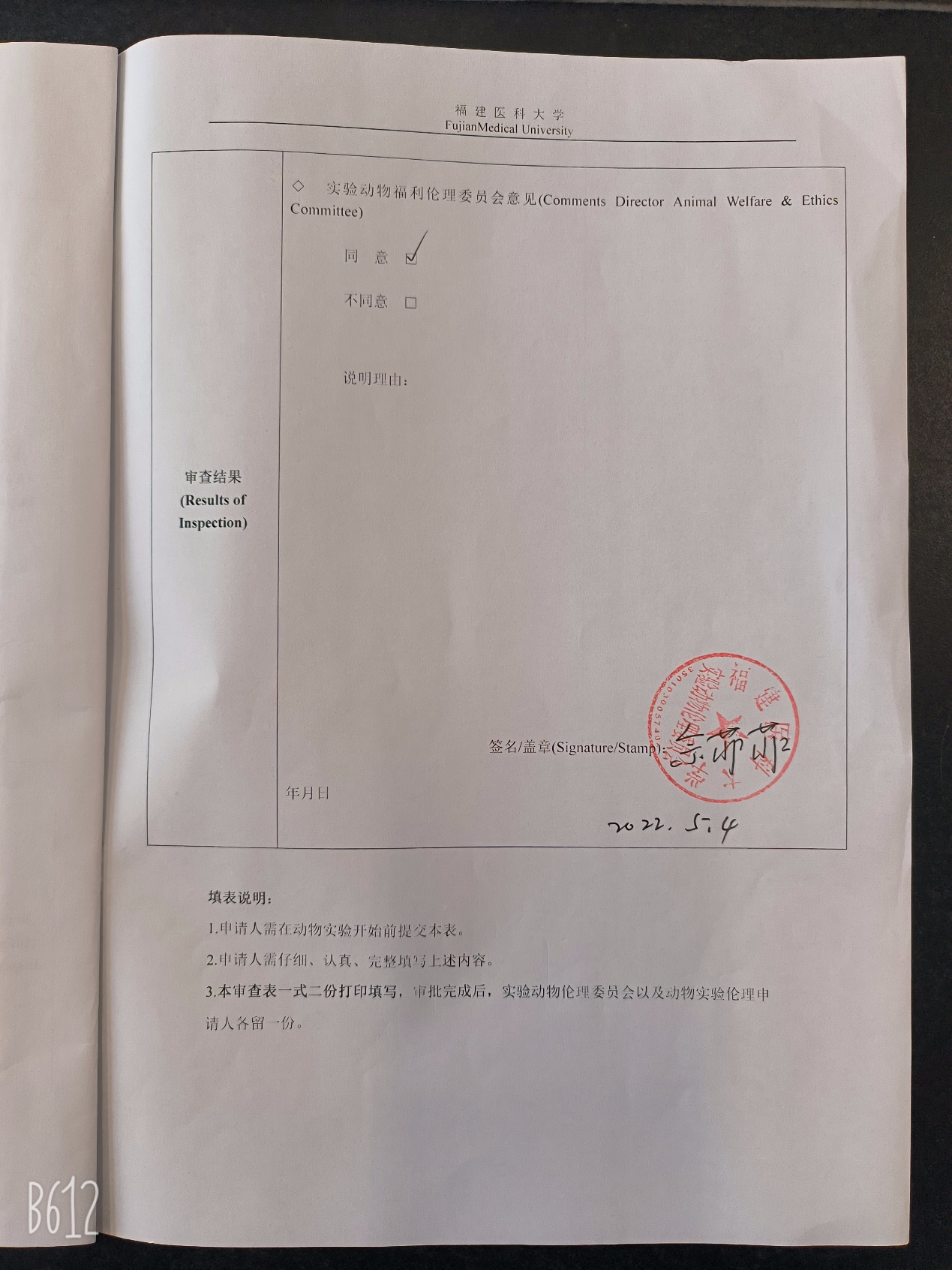


**IACUC-6**
